# Impact of Facultative Bacteria on the Metabolic Function of an Obligate Insect-Bacterial Symbiosis

**DOI:** 10.1128/mBio.00402-20

**Published:** 2020-07-14

**Authors:** Frances Blow, Nana Y. D. Ankrah, Noah Clark, Imhoi Koo, Erik L. Allman, Qing Liu, Mallappa Anitha, Andrew D. Patterson, Angela E. Douglas

**Affiliations:** a Department of Entomology, Cornell University, Ithaca, New York, USA; b Department of Veterinary and Biomedical Sciences, The Pennsylvania State University, University Park, Pennsylvania, USA; c Department of Molecular Biology and Genetics, Cornell University, Ithaca, New York, USA; National Institute of Advanced Industrial Science and Technology; University of Hawaii at Manoa

**Keywords:** *Buchnera*, *Hamiltonella*, histidine, metabolism, symbiosis

## Abstract

Beneficial microorganisms associated with animals derive their nutritional requirements entirely from the animal host, but the impact of these microorganisms on host metabolism is largely unknown. The focus of this study was the experimentally tractable tripartite symbiosis between the pea aphid Acyrthosiphon pisum, its obligate intracellular bacterial symbiont *Buchnera*, and the facultative bacterium *Hamiltonella* which is localized primarily to the aphid hemolymph (blood). Metabolome experiments on, first, multiple aphid genotypes that naturally bear or lack *Hamiltonella* and, second, one aphid genotype from which *Hamiltonella* was experimentally eliminated revealed no significant effects of *Hamiltonella* on aphid metabolite profiles, indicating that *Hamiltonella* does not cause major reconfiguration of host metabolism. However, the titer of just one metabolite, 5-aminoimidazole-4-carboxamide ribonucleotide (AICAR), displayed near-significant enrichment in *Hamiltonella*-positive aphids in both metabolome experiments. AICAR is a by-product of biosynthesis of the essential amino acid histidine in *Buchnera* and, hence, an index of histidine biosynthetic rates, suggesting that *Buchnera*-mediated histidine production is elevated in *Hamiltonella*-bearing aphids. Consistent with this prediction, aphids fed on [^13^C]histidine yielded a significantly elevated ^12^C/^13^C ratio of histidine in *Hamiltonella-*bearing aphids, indicative of increased (∼25%) histidine synthesized *de novo* by *Buchnera*. However, *in silico* analysis predicted an increase of only 0.8% in *Buchnera* histidine synthesis in *Hamiltonella*-bearing aphids. We hypothesize that *Hamiltonella* imposes increased host demand for histidine, possibly for heightened immune-related functions. These results demonstrate that facultative bacteria can alter the dynamics of host metabolic interactions with co-occurring microorganisms, even when the overall metabolic homeostasis of the host is not substantially perturbed.

## Abstract (precis)

Although microbial colonization of the internal tissues of animals generally causes septicemia and death, various animals are persistently associated with benign or beneficial microorganisms in their blood or internal organs. The metabolic consequences of these persistent associations for the animal host are largely unknown. Our research on the facultative bacterium *Hamiltonella*, localized primarily to the hemolymph of pea aphids, demonstrated that although *Hamiltonella* imposed no major reconfiguration of the aphid metabolome, it did alter the metabolic relations between the aphid and its obligate intracellular symbiont, *Buchnera*. Specifically, *Buchnera* produced more histidine in *Hamiltonella-*positive aphids to support both *Hamiltonella* demand for histidine and *Hamiltonella*-induced increase in host demand. This study demonstrates how microorganisms associated with internal tissues of animals can influence specific aspects of metabolic interactions between the animal host and co-occurring microorganisms.

## INTRODUCTION

The function of beneficial microorganisms is traditionally interpreted in terms of one or a few well-defined services that enhance host fitness. For animal hosts, frequently reported services are promotion of host nutrition and protection against natural enemies (1). These services include microbial nutrient provisioning (e.g., B vitamins, essential amino acids), degradation of dietary plant polysaccharides that are intractable to host digestion, production of toxins that are active against pathogens, and promotion of host immunological defenses (2–7). Nevertheless, there is increasing evidence that beneficial microorganisms have pervasive effects on their hosts, influencing multiple physiological systems. Many of these effects cannot be explained adequately by formally described microbial services (8–11). For many associations, this functional complexity is compounded by two further factors: (i) a high diversity and variable composition of the microbial partners (12–15) and (ii) variable contributions of different microorganisms and among-microbe interactions to microbe-dependent host traits (16–19).

The basis of this study is that the bacterial symbiosis in the pea aphid Acyrthosiphon pisum offers a superb system to investigate host interactions with beneficial microorganisms for two reasons. The first is that the association is naturally of low diversity. All pea aphids bear a bacterial symbiont, Buchnera aphidicola (gammaproteobacteria, henceforth known as *Buchnera*), which is localized to specialized insect cells known as bacteriocytes (20). In addition, many pea aphids bear one or more additional facultative bacteria, generically known as secondary symbionts, which are localized to the hemolymph as well as aphid cells (21, 22). Both *Buchnera* and these facultative symbionts are vertically transmitted via the insect ovary, but they confer different services. *Buchnera* provides the insect with essential amino acids (EAAs), required for sustained growth and reproduction on the EAA-deficient diet of phloem sap (23, 24), while some facultative symbionts confer ecologically important benefits, including protection against parasitic wasps and fungal pathogens (25). The pea aphid gut generally bears minimal numbers of transient microorganisms (26–28).

The second valuable trait of the aphid-bacterial symbiosis is that several facultative symbionts are amenable to experimental manipulation. Most research has focused on Hamiltonella defensa (gammaproteobacteria, henceforth known as *Hamiltonella*), which can be eliminated from aphids by selective antibiotic treatment, administered to *Hamiltonella*-free aphids by feeding or injection, and can be cultured in insect cell cultures and cell-free medium (29–32). Many *Hamiltonella* strains confer aphid resistance against parasitic wasps, likely mediated by toxins coded by a prophage on the genomes of protective *Hamiltonella* strains (32–35). Other data, however, suggest that *Hamiltonella* may also influence host immunity and feeding behavior (36–38). Metabolite exchange between the whitefly Bemisia tabaci and the sister taxon of aphid *Hamiltonella* (also known as *Hamiltonella defensa*) has been inferred from genomic data and metabolic modeling (39–42), but the impact of *Hamiltonella* on aphid metabolism has not, to our knowledge, been investigated systematically.

Our specific purpose was to identify how *Hamiltonella* influences the metabolic function of the pea aphid and its obligate bacterial symbiont *Buchnera*. Initial comparisons of the metabolome of aphid lines that naturally bear or lack *Hamiltonella* and a single aphid genotype with or without *Hamiltonella* led us to focus on *Buchnera*-mediated synthesis of one EAA, histidine. Using metabolism experiments and metabolic modeling, we demonstrate that for the single genotype tested, *Buchnera* synthesizes histidine at elevated rates in aphids bearing *Hamiltonella* and that both the population of *Hamiltonella* and aphid host contribute to the increased demand for *Buchnera*-derived histidine.

## RESULTS

### Metabolite profiles of pea aphids bearing and lacking *Hamiltonella*.

**FIG 1:**
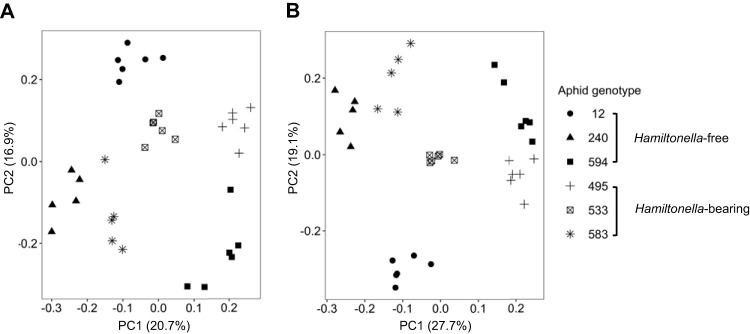
Principal-component analysis (PCA) of aphid metabolites quantified using untargeted liquid chromatography-mass spectrometry (LC-MS). (A) Positive mode. (B) Negative mode. Individual points represent biological replicates, and each symbol corresponds to an aphid genotype. The variance explained by each principal component axis is shown in parentheses.

The metabolite profile of six pea aphid genotypes, three of which were naturally infected with *Hamiltonella* and three of which were naturally *Hamiltonella*-free, was measured by untargeted liquid chromatography-mass spectrometry (LC-MS) (see Data Sets S1 and S2 in the supplemental material). Principal-component analysis (PCA) of all detected features revealed clustering by aphid genotype, but not *Hamiltonella* infection status (Fig. 1). Initial analysis with individual *t* tests, as is standard for analysis of metabolomics data (43), identified 11 metabolites that differed significantly between *Hamiltonella*-bearing and *Hamiltonella*-free aphids (see Table S1 in the supplemental material). However, when these 11 metabolites were taken for more rigorous analysis that included the effect of aphid genotype and correction for multiple testing, no metabolites differed significantly between *Hamiltonella*-bearing and *Hamiltonella*-free aphids (Table S1).

10.1128/mBio.00402-20.2 TABLE S1 Metabolites enriched in three naturally *Hamiltonella-*bearing compared to three naturally *Hamiltonella*-free aphid genotypes. Significant differences in metabolite abundances were detected using *t* tests and linear mixed-effects models. *F* statistics and *P* values are from the linear mixed-effects models where *Hamiltonella* status was treated as a fixed effect and aphid genotype was treated as a random effect. Benjamini-Hochberg (B to H) multiple testing corrections were performed on *P* values for fixed and random effects in the linear mixed-effects models. Download Table S1, DOCX file, 0.02 MB. Copyright © 2020 Blow et al. 2020 Blow et al. This content is distributed under the terms of the Creative Commons Attribution 4.0 International license.

### Construction of isogenic lines bearing and lacking *Hamiltonella*.

Two factors may contribute to the lack of significant metabolic differences between *Hamiltonella*-bearing and *Hamiltonella-*free aphid genotypes: *Hamiltonella* may have a minimal effect on the metabolite pools of the aphids; or the influence of *Hamiltonella* on aphid metabolite profiles may be obscured by metabolic variation among the aphid-*Buchnera* genotypes. To control for genotype effects, we selectively eliminated *Hamiltonella* from aphids of genotype SC_583, yielding SC_583^H-^ lacking *Hamiltonella*. (SC_583 was selected because it is amenable to *Hamiltonella* clearance using antibiotics, and it is one of the genotypes used in the initial metabolomics study.) Neither aphid performance nor the abundance and activity of their *Buchnera* populations differed significantly between the two lines (Table 1; see also Text S1A and B in the supplemental material). These data indicate that any metabolic differences between the two lines are likely direct effects of *Hamiltonella* on the metabolic function of the symbiosis, rather than a nonspecific consequence of *Hamiltonella* effects on aphid growth, development, or reproductive output or on the *Buchnera* population.

**TABLE 1:** Performance and *Buchnera* symbiosis in isogenic pea aphid lines bearing *Hamiltonella* (SC_583) and experimentally deprived of *Hamiltonella* (SC_583^H-^)^*a*^

Aphid line	Aphid performance		*Buchnera* population^*b*^	
	Intrinsic rate of increase (*r_m_*) (aphids aphid^−1^ day^−1^) (10 replicates)	Larval relative growth rate (mg mg^−1^ day^−1^) (15 replicates)	Abundance (16S copies in gDNA/aphid *ef1α* copies) (6 replicates)	Activity (16S copies in cDNA/gDNA) (6 replicates)
SC_583	0.351 ± 0.005	0.394 ± 0.006	0.77 + 0.035	1.24 + 0.060
SC_583^H-^	0.347 ± 0.010	0.377 ± 0.007	0.82 + 0.040	1.29 + 0.043

	*t*_18_ = 0.360, *P* = 0.723	*t*_28_ = 1.90, *P* = 0.068	*t*_10_ = 0.97, *P* = 0.356	*t*_10_ = 0.58, *P* = 0.574

a The last row of the table shows the *t* value (test statistic with degrees of freedom indicated as subscript) and *P* value comparing the values for the SC_583 and SC_583^H-^ aphid lines.

b Determined by quantitative PCR (qPCR), described in Text S1A in the supplemental material. gDNA, genomic DNA.

10.1128/mBio.00402-20.9 TEXT S1 Supplemental text. (A) Quantitative PCR (qPCR) analysis of *Buchnera* abundance and activity. (B) Estimation of biomass ratio of *Buchnera*/*Hamiltonella.* (C) Preparation of aphid samples for metabolomics analysis of aphid genotypes bearing and lacking *Hamiltonella.* (D) Preparation of aphid samples for metabolomics analysis of isogenic lines SC_583 and SC_583^H-^. (E) Preparation of protein hydrolysates for analysis of metabolism of dietary [^13^C_6_]histidine by isogenic aphid lines SC_583 and SC_583^H-^. (F) Diagnostic PCR for detection of *Hamiltonella*. (G) Metabolic model constraints and analysis. Download Text S1, DOCX file, 0.02 MB. Copyright © 2020 Blow et al. 2020 Blow et al. This content is distributed under the terms of the Creative Commons Attribution 4.0 International license.

### Metabolic profile of isogenic aphids bearing and lacking *Hamiltonella*.

To investigate the metabolic traits of the aphid lines SC_583 and SC_583^H-^, we applied LC-MS to measure the metabolite profiles of the 7-day-old aphid larvae that had been reared on chemically defined diets from day 2 (Table S2). Three metabolites, all with roles in phenylalanine metabolism differed significantly between the two lines after correction for multiple tests: hydroxyphenylpyruvate, prephenate, and phenyllactic acid (Table S3). These metabolites had not been identified as candidates in our initial analysis of naturally *Hamiltonella-*bearing and *Hamiltonella*-free clones (Table S1).

10.1128/mBio.00402-20.3 TABLE S2 Metabolites identified by negative-mode LC-MS analysis of metabolite pools extracted from day 7 aphid larvae of isogenic lines bearing (SC_583) and lacking (SC_583^H-^) *Hamiltonella* reared on chemically defined diets. Download Table S2, DOCX file, 0.03 MB. Copyright © 2020 Blow et al. 2020 Blow et al. This content is distributed under the terms of the Creative Commons Attribution 4.0 International license.

10.1128/mBio.00402-20.4 TABLE S3 Differential metabolite abundance between isogenic lines bearing (SC_583) and lacking (SC_583^H-^) *Hamiltonella*. LC-MS analysis was performed on metabolite pools extracted from 7-day-old aphid larvae reared on chemically defined diets. Benjamini-Hochberg multiple testing corrections were performed on *P* values obtained from *t* tests on each metabolite. Download Table S3, DOCX file, 0.04 MB. Copyright © 2020 Blow et al. 2020 Blow et al. This content is distributed under the terms of the Creative Commons Attribution 4.0 International license.

**FIG 2:**
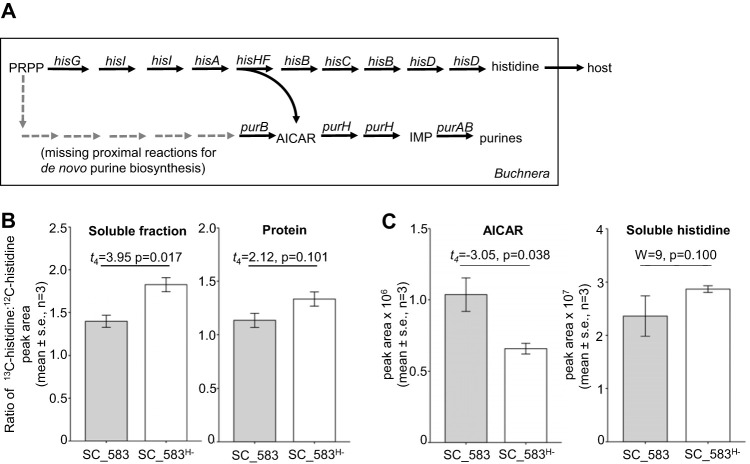
Histidine biosynthesis in isogenic aphid lines bearing *Hamiltonella* (SC_583) and lacking *Hamiltonella* (SC_583^H-^). (A) AICAR is a metabolic by-product of histidine biosynthesis in *Buchnera*. Reactions for *de novo* purine biosynthesis are absent (dashed arrows indicate genes missing from the *Buchnera* genome), and purines are instead synthesized from AICAR, which is a by-product of histidine biosynthesis in *Buchnera.* Abbreviations: PRPP, phosphoribosyl pyrophosphate; 5-aminoimidazole-4-carboxamide ribonucleotide (AICAR); IMP, inosine monophosphate. (B) Incorporation of dietary [^13^C_6_]histidine, determined as the ratio of [^13^C]histidine to [^12^C]histidine into aphid soluble pools and hydrolyzed protein pools. (C) Corrected peak area of AICAR and total soluble histidine (all measurable isotopes combined) of aphids. Statistical tests applied the critical probability of 0.025, following Bonferroni correction for two tests. s.e., standard error.

Further inspection of the metabolomics data yielded just one metabolite, 5-aminoimidazole-4-carboxamide ribonucleotide (AICAR), which had a near-significant enrichment in *Hamiltonella*-bearing aphids (i.e., significant prior to adjustment for multiple tests) in both data sets (Table S1 and Table S3). AICAR is of considerable interest because its production is linked to the overproduction of the EAA histidine by *Buchnera*. Specifically, AICAR is a by-product of *Buchnera* histidine biosynthesis and, due to deletion of the proximal reactions for *de novo* purine biosynthesis, the sole *Buchnera*-derived substrate for *Buchnera* purine synthesis (Fig. 2A). It has been argued that *Buchnera* demand for AICAR to meet its purine requirements drives the overproduction of histidine, with the excess histidine delivered to the aphid host (44, 45). We hypothesized that, in *Hamiltonella*-bearing aphids, the metabolic demand for *Buchnera*-derived histidine exceeds *Buchnera* demand for purines, leading to the accumulation of AICAR.

### *Buchnera*-mediated histidine biosynthesis.

To investigate the effect of *Hamiltonella* on *Buchnera*-mediated histidine biosynthesis, we raised larvae of the lines SC_583 and SC_583^H-^ on chemically defined diet with histidine supplied exclusively as [^13^C_6_]histidine for 5 days and then measured the ^13^C/^12^C ratio of free histidine and protein-bound histidine (Table S4). [^12^C]histidine is derived from *Buchnera-*mediated synthesis (*Hamiltonella* lacks the genetic capacity to synthesize histidine [46], and reduced [^13^C]histidine/[^12^C]histidine is indicative of increased contribution of *Buchnera*-derived histidine).

10.1128/mBio.00402-20.5 TABLE S4 Histidine isotopes and AICAR identified in soluble metabolite and hydrolyzed protein pools by negative-mode LC-MS analysis of isogenic lines SC_583 and SC_583^H-^ reared on chemically defined diets containing 2mM [^13^C_6_]histidine. Download Table S4, DOCX file, 0.01 MB. Copyright © 2020 Blow et al. 2020 Blow et al. This content is distributed under the terms of the Creative Commons Attribution 4.0 International license.

Consistent with our prediction that histidine synthesis is increased in *Hamiltonella-*bearing aphids, the ^13^C/^12^C ratio in the soluble histidine pool was significantly lower in SC_583 than SC_583^H-^ aphids at *P* = 0.025 threshold (Bonferroni correction for two tests) (Fig. 2B). The equivalent data for histidine in the protein fraction showed the same trend of reduced ^13^C/^12^C in SC_583, but the effect was not significant (Fig. 2B). ^13^C was predominantly recovered from fully labeled [^13^C]histidine (His M+6), accounting for 53 to 63% of total histidine in the soluble fraction and 47 to 55% in the protein fraction, and the equivalent values for fully unlabeled [^12^C]histidine (His M+0) were 32 to 42% and 39 to 48%, respectively (see Fig. S1 in the supplemental material).

10.1128/mBio.00402-20.1 FIG S1 Relative proportions of histidine isotopes. (A) Hydrolyzed protein pools. (B) Soluble pools of histidine. Extracted from isogenic 7-day-old line SC_583 (bearing *Hamiltonella*) and SC_583^H-^ (*Hamiltonella*-free) larvae reared on diets containing [^13^C]histidine from day 2 to day 7 of larval development. Error bars show standard deviations for three biological replicates per treatment and time point. Download FIG S1, PDF file, 0.3 MB. Copyright © 2020 Blow et al. 2020 Blow et al. This content is distributed under the terms of the Creative Commons Attribution 4.0 International license.

Further analysis of this data set showed that AICAR content, but not histidine content, was significantly elevated in SC_583 relative to SC_583^H-^ (Fig. 2C), recapitulating the results of the previous experiments (Tables S1 and S3).

### The metabolic determinants of AICAR content.

We hypothesized that the relationship between increased *Buchnera* production of histidine (as revealed by the ^13^C/^12^C ratio of histidine) and increased AICAR content of aphids bearing *Hamiltonella* could be explained by the metabolic link between the synthesis of histidine and purines in *Buchnera* (Fig. 2A). Specifically, AICAR is predicted to accumulate under conditions where the total symbiosis demand for *Buchnera*-derived histidine exceeds the *Buchnera* demand for AICAR as the substrate for purines.

**FIG 3:**
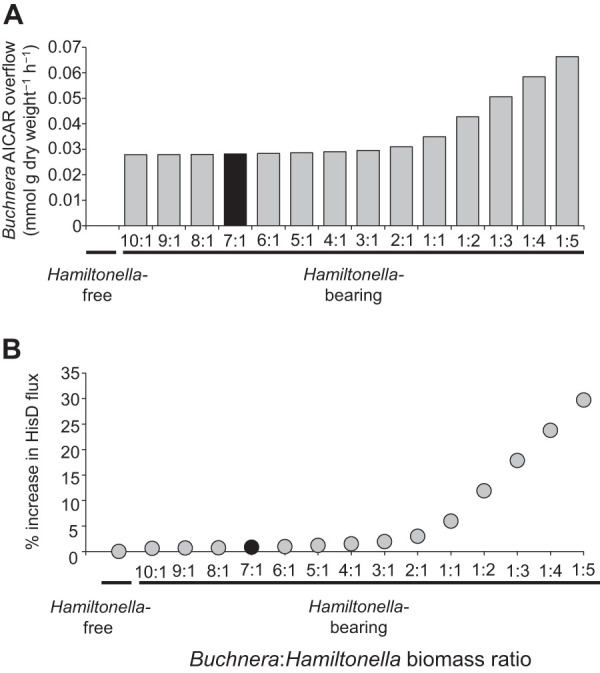
Histidine and AICAR production by *Buchnera* predicted from flux balance analysis of the *Hamiltonella*-free model comprising *Buchnera* and the aphid, and three-compartment models with increasing biomass of *Hamiltonella.* (A) AICAR overflow. (B) Flux through HisD reaction, as an index of total histidine production. Values corresponding to the empirically determined *Buchnera*/*Hamiltonella* ratio are indicated by a black bar and black circle.

To investigate whether *Hamiltonella* demand for extra histidine creates an overflow of AICAR from *Buchnera*, we compared the metabolic flux in a two-compartment metabolic model, comprising *Buchnera* and the aphid host, and three-compartment models that also included *Hamiltonella* with *Buchnera*/*Hamiltonella* biomass ratios ranging from 10:1 to 1:5 (Fig. 3). We applied flux balance analysis to quantify how AICAR production varies with histidine synthesis, as determined by flux through the HisD reaction. In the *Hamiltonella-*free model, *Buchnera* releases no AICAR (Fig. 3A). In the three-compartment model with *Buchnera*/*Hamiltonella* biomass ratio greater than one, *Buchnera* exhibits modest increase in histidine production (Fig. 3B), supporting *Hamiltonella* demand for this EAA, accompanied by AICAR overflow (Fig. 3A). As the *Hamiltonella* biomass exceeds that of *Buchnera*, the model predicts further increase in *Buchnera* histidine production and AICAR overflow. At the most extreme *Buchnera*/*Hamiltonella* biomass ratio tested of 1:5 (equivalent to 1:130 cell number ratio), HisD flux is increased by 35% and AICAR overflow is more than doubled.

The relative cell number of *Buchnera*/*Hamiltonella* in line SC_583 was determined empirically, at 1:3.9 (standard error [s.e.] = 0.3, *n* = 18), equivalent to the biomass ratio of 6.7:1 (Text S1B). Under these conditions, the predicted increase in histidine biosynthesis to support the *Hamiltonella* population is 0.8%, and the predicted AICAR overflow is 0.03 mmol g *Buchnera* biomass^−1^ h^−1^ (Fig. 3).

The modest increase in histidine yield (0.8%) predicted by our models compared to the 25% increase in histidine pools observed empirically suggests that an increase in *Hamiltonella* demand for histidine alone may not fully account for the increased *Buchnera* histidine production observed in *Hamiltonella*-bearing line SC_583. Altogether, our empirical and modeling data indicate that *Hamiltonella* induces additional demands for *Buchnera* histidine production by a third player, the aphid host.

## DISCUSSION

Animals that naturally house a few microbial taxa are powerful systems to investigate the metabolic interactions among microbial taxa and the host. Here, we leveraged the tripartite symbiosis between the pea aphid, its obligate nutritional symbiont *Buchnera*, and a facultative defensive symbiont *Hamiltonella* to investigate how *Hamiltonella* affects host-*Buchnera* metabolic function. We demonstrated that, in the presence of *Hamiltonella*, *Buchnera* increases production of the EAA histidine, and we inferred that the extra *Buchnera*-derived histidine meets both *Hamiltonella* demand and *Hamiltonella*-induced increase in host demand for this EAA. Here, in the Discussion, we explore, in turn, how our three approaches, metabolomics, metabolic experiments, and metabolic modeling, contribute to our understanding of the metabolic consequences of *Hamiltonella* and some implications for metabolic interactions between other symbiotic microorganisms and their animal hosts.

Our first metabolomics analysis revealed a far greater effect of aphid genotype than *Hamiltonella* on the metabolome of aphids naturally bearing and lacking *Hamiltonella* (Fig. 1). These results show that our methodology was appropriate to detect substantial metabolomics differences and suggest that *Hamiltonella* may not cause a major reconfiguration of the host metabolome. Additionally, the clustering of the metabolomics data by genotype is indicative of large-scale intraspecific variation in metabolic function of the pea aphid. This striking pattern complements published evidence for significant among-genotype variation in aphid utilization of sucrose and amino acids (29, 47–49), the chief carbon and nitrogen sources in the aphid diet of plant phloem sap. Furthermore, this variation has been causally linked to specific genes of the aphid and *Buchnera*, as well as variation in *Buchnera* population size (47, 50–52). These patterns are also fully consistent with evidence from other animals that host genotype can strongly influence metabolic traits linked to microbiome function (53–57).

Although *Hamiltonella* does not perturb the global metabolic homeostasis of its aphid host, our metabolomics data sets included one metabolite, AICAR, with elevated titer in *Hamiltonella-*bearing aphids for both the comparisons between genotypes that naturally harbor and lack *Hamiltonella* and between a single genotype bearing and experimentally deprived of *Hamiltonella*. The correspondence between the presence of *Hamiltonella*, increased AICAR, and increased *Buchnera*-mediated histidine production, as determined by our ^13^C metabolic experiments, confirmed our hypothesis that the additional AICAR was likely of *Buchnera* origin and linked to increased demand for *Buchnera*-derived histidine in *Hamiltonella*-bearing aphids.

Aphids bearing *Hamiltonella* are expected to have an elevated histidine requirement because *Hamiltonella* is auxotrophic for this EAA (46, 58). *Hamiltonella* has the genetic capacity to synthesize just two EAAs, threonine and lysine, and it is expected to be a sink for the other eight EAAs (including histidine), all of which are synthesized by *Buchnera* (24). These predicted EAA fluxes from *Buchnera* to *Hamiltonella* do not translate into significant effects on the titers of histidine or other EAAs in the aphid metabolome, presumably because of homeostatic controls over metabolite pool sizes. *Hamiltonella* may impose a greater demand for histidine than other EAAs; alternatively, increased flux through other *Buchnera* EAA biosynthetic pathways in *Hamiltonella*-bearing aphids may have gone undetected in our study because it did not result in the accumulation of unique by-product(s) equivalent to AICAR for histidine synthesis. An indication that *Hamiltonella* may alter the metabolism of a second EAA, phenylalanine, comes from the significant underrepresentation of three intermediates in the phenylalanine biosynthetic and catabolic pathways (hydroxyphenylpyruvate, prephenate and phenyllactic acid) in *Hamiltonella*-bearing isogenic aphids (see Table S3 in the supplemental material).

Interestingly, the nutritional requirements of *Hamiltonella* resulted in no discernible reduction of aphid fitness, despite increased EAA demand. Possible contributory factors were that we used naturally occurring aphid-*Hamiltonella* combinations and a susceptible plant cultivar for insect culture. In published studies, the effect of *Hamiltonella* on aphid performance varies with aphid and bacterial genotype (59–61) and can be particularly deleterious for aphids reared on partially resistant plants (36, 62).

An important inference from this study is that *Hamiltonella* demand for histidine is unlikely to account fully for the difference in histidine production between *Hamiltonella-*bearing and *Hamiltonella*-free lines. The chief evidence came from metabolic models that assumed fixed aphid demand for histidine; when *Hamiltonella* at the empirically determined biomass was added to the aphid-*Buchnera* model, the computed flux of histidine synthesis increased by just 0.8%, substantially less than the observed 25% difference in *Buchnera*-derived histidine production between aphids bearing and lacking *Hamiltonella.* A possible factor contributing to this discrepancy may have been the simplifying assumptions required to construct the flux balance models (63, 64), although the model equations are not discernibly biased to underestimate *Hamiltonella* demand for histidine. It is most probable that increased host demand for histidine in *Hamiltonella*-bearing aphids contributes much of the discrepancy between the empirical data (Fig. 2B) and model data (Fig. 3).

Why might *Hamiltonella* increase host demand for histidine and possibly other *Buchnera-*derived EAAs? Two processes may be involved. First, *Hamiltonella* has been demonstrated to alter the cellular immunity of pea aphids, specifically by increasing the population of hemocytes (37). This effect would increase the host sink for histidine because as for animals generally (65, 66), immune cell proliferation in aphids is metabolically demanding and requires metabolic resources, including EAAs. Second, aphid feeding, including probing behavior and food ingestion can be altered by *Hamiltonella* (36). These feeding traits can substantially affect dietary EAA supply to the aphid by influencing aphid choice of feeding site and food consumption rates. Previous research has demonstrated that rearing aphids on diets lacking a specific EAA results in increased synthesis of that EAA by *Buchnera* (67). This suggests that increased production of histidine by *Buchnera* could arise from feeding changes in aphids bearing *Hamiltonella* that resulted in reduced uptake of dietary histidine.

In conclusion, this study has revealed that the tripartite relationship between two bacterial symbionts and their aphid host is metabolically interactive. Specifically, the nutritional requirements of one bacterium, *Hamiltonella*, can modify the metabolic function of a second symbiont, *Buchnera*, for increased histidine production without altering the *Buchnera* population size, and the *Buchnera* response to support *Hamiltonella* is likely compounded by the *Hamiltonella*-induced increase in host demand for histidine. These findings raise two general questions. The first relates to the role of *Hamiltonella* as a defensive symbiont that protects the aphid host against parasitic wasps (33). Although this defensive function has been attributed to toxins encoded by a prophage on the *Hamiltonella* genome (35), future research should consider the possible contribution to parasitoid resistance of *Hamiltonella*-induced changes to aphid metabolism and immunity. The second question is the incidence of multiway metabolic interactions in symbioses. On the one hand, the substantial among-genotype differences in the aphid metabolome identified in this study suggests that further insights can be gleaned from analysis of intraspecific variation in these interactions. This avenue would be a productive extension of recent research on variation in aphid-*Buchnera* interactions (50, 52) and idiosyncratic effects of facultative symbionts on host phenotype (59–61). On the other hand, the broad principle of multiway interactions may be general to multiple taxa localized to the hemolymph and cells of insects (68), including other facultative symbionts of aphids and bacteria with a broad distribution in arthropods, e.g., *Wolbachia*, *Spiroplasma*. As in this study, these future investigations will be facilitated by the combined application of metabolomics, isotope tracer experiments, and metabolic modeling.

## MATERIALS AND METHODS

### Experimental aphids.

The experiments were conducted on six genotypes of the pea aphid *Acyrthosiphon pisum* collected from an alfalfa field in Ithaca, NY, USA, in May 2015. All genotypes bore the vertically transmitted bacterial symbiont *Buchnera*; genotypes SC_12, SC_240 and SC_594 bore no previously described facultative symbionts, also known as secondary symbionts, SC_533 and SC_583 bore *Hamiltonella*, and SC_495 had both *Hamiltonella* and *Spiroplasma* (50).

The aphids were maintained on Vicia faba cv. Windsor at 20°C with 16-h light/8-h dark light cycle. To generate age-synchronized larvae, adult apterous females were allowed to larviposit for 24 h on V. faba plants or excised leaves and then removed. The deposited larvae were left to develop for a further day (to day 2) for use in experiments.

### Metabolomics (LC-MS) analysis.

To analyze aphid genotypes that naturally harbored and lacked *Hamiltonella*, five or six replicate pools of mixed-age aphids of each genotype were collected from routine culture on plants, with 25 mg fresh weight per replicate. Following sample preparation (see Text S1C in the supplemental material), 5 μl of each sample was injected onto an AB SCIEX 5600 TripleTOF (triple time of flight) liquid chromatography-mass spectrometry (LC-MS) for analysis in positive and negative electrospray ionization (ESI) mode, with method blanks and a pooled quality control (QC) sample. Samples were separated by reverse-phase high-performance liquid chromatography (HPLC) using Prominence 20 UFLCXR system (Shimadzu) with a BEH C_18_ column (100 mm × 2.1 mm; 1.7 μm particle size; Waters) maintained at 55°C and a 20-min aqueous acetonitrile gradient (flow rate 250 μl min^−1^). The initial conditions were 97% solvent A (HPLC grade water with 0.1% formic acid) and 3% solvent B (HPLC grade acetonitrile with 0.1% formic acid), increasing to 45% solvent B at 10 min, 75% solvent B at 12 min where it was held until 17.5 min before returning to initial conditions. The eluate was delivered into a 5600 (QTOF) TripleTOF using a Duospray ion source (AB SCIEX). The capillary voltage was set at 5.5 kV in positive ion mode and 4.5 kV in negative ion mode, with declustering potential of 80 V. The mass spectrometer was operated in information-dependent acquisition mode with 100-ms survey scan from 100 to 1200 *m/z*, and up to 20 MS/MS (tandem MS) product ion scans (100 ms) per duty cycle using a collision energy of 50 V with a 20-V spread.

Instrument raw data files were converted into mzML format using Proteowizard (69) and analyzed using MS-DIAL (70). Mass spectrometry tolerances for MS1 and MS2 were set to 0.01 Da and 0.05 Da, corresponding to the resolution of the TripleTOF instrument. For smoothing extracted-ion chromatography, the linear weighted moving average was applied with a smoothing level of 3, and the minimum peak height was set to 3,000 for noise signal. Compound identification used MS/MS similarity to the curated public library in MS-DIAL with 80% similarity threshold and peak areas normalized using the internal standard chlorpropamide (Santa Cruz Biotech).

Metabolomics analysis of isogenic lines SC_583 and SC_583^H-^ used three groups of 10 2-day-old larvae administered diet with 2 mM histidine to mimic *V. faba* phloem sap (71). Five days later, the larvae (7-days-old, approximately 25 mg fresh aphid material per sample) were snap-frozen in liquid nitrogen and stored at –80°C. Following extraction of metabolites (protocol in Text S1D), metabolites were separated using a Waters XSelect HSS T3 column (100 Å, 5 μm, 2.1 mm × 100 mm) fitted with a Restex UltraShield 0.2-μm precolumn filter and analyzed on a Thermo Exactive Plus Orbitrap (72–74). Blanks and a pooled QC sample were included in the analysis. Raw data files were converted to mzXML format and processed in MAVEN (75) using an in-house targeted metabolite library (72). Compounds were identified based on *m*/*z* (±10 ppm) and retention time (±0.5 min) tolerances to standards. Peak areas were normalized against the total ion chromatogram to account for analytical drift, followed by blank subtraction.

For analysis of dietary [^13^C]histidine metabolism by isogenic aphid lines SC_583 and SC_583^H-^, six replicate groups of 2-day-old aphids per line were raised on diets containing 2 mM ^13^C_6_-labeled histidine for 5 days (to day 7). Each replicate, comprising 30 larvae (approximately 25 mg fresh aphid material), was snap-frozen in liquid nitrogen and stored at –80°C. Three replicates per line were analyzed for soluble metabolites and extracted as described above, and protein hydrolysates were prepared as described previously (76) (Text S1E). Samples were analyzed on a Thermo Exactive Plus Orbitrap as described above. For histidine isotopes, all isotopic peaks were picked manually using the expected isotopic mass and the tolerances *m*/*z* (±10 ppm) and retention time (±0.5 min).

### Antibiotic treatment of pea aphid genotype SC_583 to eliminate *Hamiltonella*.

The larvae of genotype SC_583 were treated with antibiotics as in reference 29. Three cages, each with five 2-day-old larvae of genotype SC_583, were maintained on a chemically defined diet (formulation in reference 77 with 0.5 M sucrose and 0.15 M amino acids), supplemented with the antibiotics gentamicin, cefotaxime, and ampicillin (Sigma) each at 50 μg ml**^−^**^1^ diet for 5 days (to day 7), when they were transferred individually to plants and allowed to larviposit.

A single offspring (generation 2) per aphid was tested for *Hamiltonella* by diagnostic PCR assay (50) (Text S1F). Up to 10 siblings of individuals that tested negative were transferred individually to fresh plants, and five of their progeny (generation 3) were tested by PCR for *Hamiltonella*. This was repeated to generation 10. Where an aphid tested positive for *Hamiltonella*, all codescendants from the generation 2 aphid were discarded. By this procedure, we generated the *Hamiltonella*-free line SC_583^H-^. As expected, hemolymph samples from leg bleeds of genotype SC_583 bore many bacterial cells of the morphology predicted for *Hamiltonella* (21, 31,) but hemolymph samples from line SC_583^H-^ were bacteria free.

### Aphid performance assays.

To determine larval relative growth rate (RGR) of aphid lines SC_583 and SC_583^H-^, 15 replicate groups of five 2-day-old aphids were weighed (±1 μg) and confined to clip cages on *V. faba* plants. On day 7, the surviving larvae in each clip cage were counted and weighed. RGR was calculated as log*_e_* (day 7 weight per aphid/day 2 weight per aphid)/5 days. To determine the intrinsic rate of increase (*r_m_*), larvae deposited by adults over 24 h on *V. faba* plants were allowed to develop for 6 days, when they were in the final larval stadium, and then individually transferred to the underside of a leaf of a fresh plant in a clip cage. Aphids were then examined daily until the first offspring was deposited, to give the time from larviposition to onset of reproduction (*d*). Progeny were subsequently counted and removed every several days until 2*d*, yielding the total number of progeny per aphid (*Md*). The formula *r_m_* = 0.745(log*_e_ M_d_*)/*d* (78) was applied, with 10 replicate individuals per line.

### Metabolic model reconstruction and analysis.

A genome scale metabolic model of Hamiltonella defensa was generated by combining two draft model reconstructions. The first identified gene orthologs in the *Hamiltonella defensa* genome (NCBI JAABOV000000000) and Escherichia coli strain K-12 substrain MG1655 by reciprocal BLAST searches and then manually extracted reactions encoded by these genes from E. coli strain K-12 substrain MG1655 metabolic model iML1515 (79) to create a draft model, as previously described (41). The second draft reconstruction was generated from the automated reconstruction pipeline ModelSEED (80) using a RAST (81) reannotated *Hamiltonella defensa* genome as input. The two draft models were then integrated and manually curated to remove redundant reactions and ensure correct reaction gene association, directionality, stoichiometry, and mass-charge balance. *Hamiltonella*-specific features and genes encoding metabolic reactions absent in the E. coli iML1515 metabolic model were identified by literature review and searches of the BioCyc, KEGG, EcoCyc, BiGG, and BRENDA databases (82–86) and then added to the draft model.

The *Buchnera* metabolic model was updated from the published model (44, 87) by removing two reactions, adding 62 reactions (Data Set S3A) and updating the biomass equation (see Data Set S3B for details).

10.1128/mBio.00402-20.6 DATA SET S1 Positive-mode LC-MS analysis of six aphid genotypes naturally bearing and lacking *Hamiltonella.* (A) Metabolites identified by fragmentation of precursor ions (MS2). (B) Metabolites identified from precursor ions (MS1). (C) Precursor ions that could not be identified as known metabolites. (D) Sample key. Download Data Set S1, XLSX file, 1.2 MB. Copyright © 2020 Blow et al. 2020 Blow et al. This content is distributed under the terms of the Creative Commons Attribution 4.0 International license.

10.1128/mBio.00402-20.7 DATA SET S2 Negative-mode LC-MS analysis of six aphid genotypes naturally bearing and lacking *Hamiltonella.* (A) Metabolites identified by fragmentation of precursor ions (MS2). (B) Metabolites identified from precursor ions (MS1). (C) Precursor ions that could not be identified as known metabolites. (D) Sample key. Download Data Set S2, XLSX file, 1.0 MB. Copyright © 2020 Blow et al. 2020 Blow et al. This content is distributed under the terms of the Creative Commons Attribution 4.0 International license.

10.1128/mBio.00402-20.8 DATA SET S3 Metabolic model data. (A) Updates to published *Buchnera* model iSM199. (B) Objective function components for *Buchnera*, *Hamiltonella*, and aphid metabolic models. (C) Aphid hemolymph growth medium used for simulations. Download Data Set S3, XLSX file, 0.02 MB. Copyright © 2020 Blow et al. 2020 Blow et al. This content is distributed under the terms of the Creative Commons Attribution 4.0 International license.

A genome scale metabolic model for the aphid host was generated as previously described (41), using aphid reactions involved in primary metabolism identified from publicly available *Acyrthosiphon pisum* genome data (NCBI: GCA_000142985.2). Additional reactions to generate or consume dead-end metabolites were identified and incorporated into the aphid host draft reconstruction. Individual bacterial and host metabolic models were integrated into a three-compartment model using previously described methods (41, 88). Model testing was conducted in COBRA Toolbox version 3.0 (89) run in Matlab 2015b (The MathWorks Inc.), using the Gurobi version 6.5.0 solver (Gurobi Optimization 2016). Details of model analysis and constraints applied are provided in Text S1G.

### Statistical analyses.

All statistical analyses were performed in R (90). Data sets were inspected for normality and homoscedasticity using Shapiro-Wilk and Kolomogorov-Smirnov tests and Bartlett’s test, respectively. Differences in data with normal distributions and homogenous variances were tested with Student’s *t* test, and data sets with nonnormal distributions and/or heterogeneous variances were tested with Mann-Whitney U test. Statistically significant differences in metabolite abundance detected by LC-MS analysis of six aphid genotypes were calculated using a linear mixed-effects model (LMM) using the “lmer” function in the lme4 package, version 1.1-19 (91). *Hamiltonella* status was treated as a fixed effect, and aphid genotype was treated as a random effect. Residuals were visually inspected for normality, and an analysis of variance (ANOVA) was performed using the car package, version 3.0-2 (92). *P* values were adjusted for multiple testing using the Benjamini-Hochberg method.

### Data availability.

The *Hamiltonella defensa* genome assembly and raw sequencing reads used to generate the genome scale metabolic model are available in the GenBank repository under accession number PRJNA602159 (https://www.ncbi.nlm.nih.gov/bioproject/PRJNA602159). The multicompartment model is provided in three formats—SBML (.xml), MATLAB (.mat), and Excel (.xls)—and deposited in GitHub (https://github.com/na423/Aphid_symbiosis). An SBML file of the models is also available in the BioModels database (93) with the identifier MODEL2001310002. All other data are provided in supplemental files.
