# Supplementary material for: Impact of Facultative Bacteria on the Metabolic Function of an Obligate Insect-Bacterial Symbiosis
Source: mBio. 2020 Jul 14;11(4):e00402-20. doi: 10.1128/mBio.00402-20 (PMC7360925; doi:10.1128/mBio.00402-20)
Supplement: TABLE S1 [file mBio.00402-20-st001.docx]

**TABLE S1** Metabolites enriched in three naturally *Hamiltonella*-bearing compared to three naturally *Hamiltonella*-free aphid genotypes. Significant differences in metabolite abundances were detected using *t*-tests and linear mixed-effects models. *F* statistics and *p*-values are from the linear mixed effects models where *Hamiltonella* status was treated as a fixed effect and aphid genotype was treated as a random effect. Benjamini-Hochberg (B-H) multiple testing corrections were performed on *p*-values for fixed and random effects in the linear mixed-effects models.

|  |  | ***t*-test (*Hamiltonella*)** | | **ANOVA fixed effect**  **(*Hamiltonella*)** | | | **ANOVA random effect**  **(aphid genotype)** | | |
| --- | --- | --- | --- | --- | --- | --- | --- | --- | --- |
| **Metabolite** | **Fold-enrichment in *Hamiltonella*-bearing aphids** | ***t*_4_** | ***p*-value** | ***F*_4_** | ***p*-value** | **B-H**  ***p*-value** | ***F*_28_** | ***p*-value** | **B-H**  ***p*-value** |
| N-Acetylglutamate | 2.883 | 3.025 | 0.005 | 4.083 | 0.113 | 0.388 | 18.567 | 0.0002 | 0.0002 |
| Glycerophosphate | 2.123 | 6.315 | 0.000 | 13.509 | 0.021 | 0.388 | 104.520 | <0.0001 | 0.0001 |
| Hirsutrin | 1.939 | 3.363 | 0.002 | 2.609 | 0.182 | 0.388 | 25.563 | <0.0001 | 0.0001 |
| Chlorogenic acid | 1.896 | 6.081 | 0.000 | 6.307 | 0.066 | 0.388 | 66.526 | <0.0001 | 0.0001 |
| 5-Aminoimidazole-4-carboxamide ribonucleotide (AICAR) | 1.671 | 2.833 | 0.008 | 1.020 | 0.370 | 0.411 | 16.212 | 0.0004 | 0.0004 |
| Scopoletin | 1.578 | 2.630 | 0.014 | 1.107 | 0.352 | 0.411 | 21.372 | 0.0001 | 0.0001 |
| Phlorizin | 1.533 | 2.671 | 0.012 | 1.320 | 0.315 | 0.411 | 29.630 | <0.0001 | 0.0001 |
| Riboflavin | 1.529 | 3.333 | 0.002 | 1.400 | 0.302 | 0.411 | 34.977 | <0.0001 | 0.0001 |
| Succinate | 1.391 | 3.997 | 0.000 | 2.917 | 0.163 | 0.388 | 108.188 | <0.0001 | 0.0001 |
| Adenosine diphosphate | 1.298 | 2.916 | 0.008 | 6.623 | 0.062 | 0.388 | 399.743 | <0.0001 | 0.0001 |
