# Supplementary material for: Impact of Facultative Bacteria on the Metabolic Function of an Obligate Insect-Bacterial Symbiosis
Source: mBio. 2020 Jul 14;11(4):e00402-20. doi: 10.1128/mBio.00402-20 (PMC7360925; doi:10.1128/mBio.00402-20)
Supplement: TEXT S1 [file mBio.00402-20-s0001.docx]

**Text S1. Supplementary Text**

**Text S1A. Quantitative PCR (qPCR) analysis of *Buchnera* abundance and activity.** Six 7-day-old aphids of SC_583 and SC_583^H-^ reared on chemically-defined diets containing 2mM histidine were tested by the procedure of (Chung et al., 2018) for *Buchnera* abundance (16S rRNA gene copy numbers normalized to aphid gene *ef1α*) and *Buchnera* activity (16S copy number in cDNA, relative to gDNA). Genomic DNA (gDNA) and total RNA were extracted from whole individual aphids in Trizol Reagent (Thermo Fisher Scientific), followed by the dual back-extraction protocol of (Triant and Whitehead, 2009). cDNA was generated from 250 ng total RNA using the Applied Biosystems High-Capacity cDNA Reverse Transcription Kit (Applied Biosystems) with random primers following manufacturer's protocol. The resulting cDNA was diluted 1000-fold for qPCR assays. qPCR reactions comprised 5 µl 2 x iQ SYBR Green Supermix (Bio-Rad), 0.5 µl primer mix (*Buchnera* 16S rRNA F: 5’- AGCGGCCTCCTAAACGAAAA -3’; R: 5’- AGTCGACATCGTTTACGGCA -3’; aphid *ef1α* F: 5’- AGAATGGACAAACCCGTGAA -3’; R: 5’- CACTGTATGGTGGTTCAGTAGAG -3’)), and 0.5 µl gDNA or cDNA in 10 µl reaction volume. qPCR assays were performed in duplicate in a CFX-Connect Real Time System (Bio-Rad) with negative controls for gDNA and RNA extraction, cDNA synthesis and qPCR reagents run alongside samples in each assay. Dissociation curves performed at the end of each assay confirmed a single gene peak and no primer dimerization. Thermal-cycling conditions were: 95^o^C for 5 min; 40 cycles of 95^o^C for 15 s followed by 60^o^C for 1 min; dissociation curve of 65^o^C for 5 s followed by temperature ramp to 95^o^C in 0.5^o^C increments.

**Text S1B. Estimation of biomass ratio of *Buchnera:Hamiltonella***

**Microscopical quantification of relative abundance of *Buchnera* and *Hamiltonella*.** Eighteen individual plant-reared 7-day-old larvae of genotype SC_583 bearing *Hamiltonella* were rinsed in filter-sterilized deionized water and hand-homogenized in 25 µl sterile PBS. Each homogenate was fixed by adding 75 µl 4% Paraformaldehyde (Sigma) and incubated at room temperature for 90 min. Cells were pelleted by centrifugation at 6000 x g for 5 min at room temperature, washed once in 100 µl sterile PBS with centrifugation and then incubated in 100 µl PBS containing Hoechst 33342 (Thermofisher) at 1 ng ml^-1^ for 30 min at room temperature in the dark. The stained cells were centrifuged at 6000 x g for 3 min at room temperature, and the pellet was resuspended in 200 µl sterile PBS. Four µl of each cell suspension was sandwiched between two glass coverslips for imaging on a Zeiss 710 confocal microscope. *Buchnera* and *Hamiltonella* cells were distinguished by morphology: *Buchnera* cells are spherical and have a diameter of approx. 3 µm, whereas *Hamiltonella* cells are rod-shaped and ca. 0.5 µm x 2 µm.

**Estimation of biomass ratio.** The relative abundance of cells of *Buchnera* and *Hamiltonella* in the aphid homogenates was 0.256:1 (s.e. 0.044 n=18). As summarized in Table S6, we used the calculated volume per cell to estimate the dry weight (equivalent to biomass) cell^-1^. From these data, we obtained an estimated relative biomass of *Buchnera:Hamiltonella* in the aphids at 6.7:1.

Table for Text S1B. Empirical estimation of *Buchnera*:*Hamiltonella* biomass ratio.

| Bacterium | Volume cell^-1^  (µm^3^)^1^ | Dry weight per cell (pg)^2^ | Relative cell number^3^ | Relative dry weight^4^ | Dry weight ratio^5^ |
| --- | --- | --- | --- | --- | --- |
| *Buchnera* | 14.14 | 1.805 | 0.256 | 0.462 | 6.7:1 |
| *Hamiltonella* | 0.39 | 0.069 | 1 | 0.069 |  |

^1^ Estimated as sphere of 3 µm diam. for *Buchnera* and cylinder of 2 µm length and 0.5 µm diam. for *Hamiltonella*

^2^ Calculated by the allometric equation linking dry weight (DW in pg) to volume (V in µm^3^) of bacterial cells: log (DW) = -0.79 X log (V) [Norland, S., Heldal, M., and Tumyr, O. (1987). On the relation between dry matter and volume of bacteria. Microbial Ecology *13*, 95-101].

^3^ Determined empirically from counts of bacterial cells in aphid homogenates

^4^ Product of dry weight per cell and relative cell number

^5^ *Buchnera:Hamiltonella*, applied as biomass ratio in the three-compartment metabolic model (Fig. 3).

**Text S1C. Preparation of aphid samples for metabolomics analysis of aphid genotypes bearing and lacking *Hamiltonella.*** Metabolites were extracted from each of 5 or 6 samples per genotype in 1 mL ice-cold 3:3:2 acetonitrile:isopropyl alcohol:water containing the internal standards alanine, pyruvate, isoleucine, glutamate, uracil, and tyrosine along with 0.1 mm zirconia/silica beads (BioSpec. Products). Samples were homogenized in a Precellys 24 homogenizer for 2x20 s per cycle, for 2 cycles at 6500 rpm, with samples placed on ice between cycles. Samples were then shaken for an additional 6 min at 4 °C. The samples were clarified by centrifugation at >14,000 x *g* and 4°C for 10 min, and the clarified supernatants were transferred to a new tube and dried in a SpeedVac (Thermo Fisher Scientific). The dried pellets were washed with 500 µL 1:1 acetonitrile:water and clarified by centrifugation at >14,000x*g* and 4 °C for 10 min. The clarified supernatants were transferred to a new tube and dried in a SpeedVac. Dried samples were resuspended in 100 µL 3% methanol:97% water containing 1 µM internal standard chlorpropamide (Santa Cruz Biotech).

**Text S1D. Preparation of aphid samples for metabolomics analysis of isogenic lines SC_583 and SC_583^H-^.** To extract metabolites, 1 mL ice-cold 80% methanol:20% water (0.1% formic acid) and 1 mm zirconia/silica beads were added to each tube. Samples were homogenized in a Precellys 24 homogenizer for two cycles at 6500 rpm, 2 x 30 seconds per cycle, with samples placed on ice between cycles. The samples were clarified by centrifugation at 14,000 x g and 4 °C for 10 minutes. The clarified supernatants were transferred to a new tube and dried in a SpeedVac (Thermo Fisher Scientific). Dried samples were resuspended to 0.286 mg µl^-1^, in 3% methanol:97% water containing 1 µM chlorpropamide (Santa Cruz Biotech). Samples were vortexed and sonicated for 5 minutes to ensure complete resuspension. Samples were then centrifuged at maximum speed (>15,000 x g) for 10 minutes to remove any insoluble material. 45µL of each was transferred to an MS vial, centrifuged at 8,900 rpm for 30 seconds, and randomized for injection.

**Text S1E. Preparation of protein hydrolysates for analysis of metabolism of dietary ^13^C-histidine by isogenic aphid lines SC_583 and SC_583^H-^.** Each aphid sample was transferred to a 2 mL glass vial and, after addition of 400 µl 6 M HCl solution (containing 0.5 µM ^13^C_4_,^15^N-aspartate), the vials were flushed with nitrogen for 30 s ﻿and immediately capped. Following incubation at 110 °C for 36 h, the reaction mixture was cooled and then transferred to 1.5 mL centrifuge tubes and centrifuged at 18,800 x g at 4°C for 10 min. The supernatant was collected, evaporated to dryness in a SpeedVac (Thermo Scientific) and dissolved in 200 µL 10 mM ammonium acetate solution with 1 µM chlorpropamide (Santa Cruz Biotech). Samples were stored at -20°C until analysis.

**Text S1F. Diagnostic PCR for detection of *Hamiltonella*.** DNA was extracted by the “salting-out” method [1] and the PCR reactions comprised 100 ng DNA, 10 µM primers (F: 5’- AGTTTGATCATGGCTCAGATTG-3’; R: 5’ AAATGGTATTSGCATTTATCG-3’ [2] and Master Mix Quick-Load Taq (New England BioLabs, Ipswich, MA), with the “Touchdown” PCR cycle: 94°C 2 min, 11 cycles of (94°C 20 sec, 56°C (declining 1°C each cycle) 50 sec, 72°C 30 sec), 25 cycles of 94°C 2 min, 45°C 50 sec, 72°C 2 min and a final extension of 72°C 5 min. The products were separated by electrophoresis in a 2% agarose gel and visualized with SYBR Safe DNA Gel Stain, to score for the predicted 471 bp band.

References for Text S1F:

1. Sunnucks P,Hales DF. 1996. Numerous transposed sequences of mitochondrial cytochrome oxidase I-II in aphids of the genus Sitobion (Hemiptera: Aphididae). Mol Biol Evol 13: 510-524.

2. Henry LM, Peccoud J, Simon JC, Hadfield JD, Maiden MJ, Ferrari J, Godfray HC. 2013. Horizontally transmitted symbionts and host colonization of ecological niches. Curr Biol 23: 1713-1717.

**Text S1G. Metabolic model constraints and analysis.** Constituents of the external medium used for simulations are provided in Table S3C. All model simulations applied aerobic conditions. To investigate the impact of *Hamiltonella* on *Buchnera*-mediated production of histidine and AICAR, flux balance analysis was first performed on the two-compartment *Hamiltonella*-free model to establish baseline flux values required for optimal aphid and *Buchnera* growth. Reaction flux values for phosphoribosyl-amino-imidazole-carboxamide formyltransferase (PurH) and ATP synthase from the *Hamiltonella*-free model simulations were applied as constraints to the three-compartment model including *Hamiltonella*, thereby maintaining *Buchnera* nucleotide production and utilization flux at the optimal levels for the *Hamiltonella*-free model. Increases in *Buchnera* histidine production were calculated as the %-change histidine production flux between the two-compartment and three-compartment models with different relative biomass of *Buchnera* and *Hamiltonella*. *Buchnera* AICAR overflow flux was obtained by adding an AICAR sink reaction to the models to capture AICAR produced in excess of the requirement for optimal *Buchnera* nucleotide synthesis.
