# Supplementary material for: Impact of Facultative Bacteria on the Metabolic Function of an Obligate Insect-Bacterial Symbiosis
Source: mBio. 2020 Jul 14;11(4):e00402-20. doi: 10.1128/mBio.00402-20 (PMC7360925; doi:10.1128/mBio.00402-20)
Supplement: TABLE S2 [file mBio.00402-20-st002.docx]

**TABLE S2** Metabolites identified by negative-mode LC-MS analysis of metabolite pools extracted from day-7 aphid larvae of isogenic lines bearing (SC_583) and lacking (SC_583^H-^) *Hamiltonella* reared on chemically-defined diets.

| **Metabolite** | **Peak area** | | | | | |
| --- | --- | --- | --- | --- | --- | --- |
|  | SC_583 | | | SC_583^H-^ | | |
|  | Rep1 | Rep2 | Rep3 | Rep1 | Rep2 | Rep3 |
| Glycine | 4710000 | 4280000 | 7840000 | 5290000 | 8700000 | 9080000 |
| Pyruvate | 274000 | 185000 | 286000 | 383000 | 514000 | 820000 |
| Alanine/Sacrosine | 23300000 | 20500000 | 45700000 | 26300000 | 42800000 | 55700000 |
| Lactate | 30900000 | 30900000 | 30900000 | 1090000 | 30900000 | 12900000 |
| Acetoacetate | 798000 | 1280000 | 2270000 | 466000 | 1150000 | 3420000 |
| 2-Oxobutanoate | 4390000 | 458000 | 6720000 | 4390000 | 4390000 | 131000 |
| 4-Aminobutyrate | 10800000 | 11600000 | 16500000 | 7560 | 18300000 | 15400000 |
| AICAR | 1060000 | 842000 | 870000 | 564000 | 741000 | 422000 |
| Serine | 10800000 | 9980000 | 16600000 | 8450000 | 15200000 | 18200000 |
| Glycerate | 20800000 | 24900000 | 24200000 | 21000000 | 28100000 | 22600000 |
| Histamine | 517000 | 559000 | 719000 | 711000 | 1240000 | 1040000 |
| Uracil | 263000 | 299000 | 503000 | 306000 | 526000 | 643000 |
| Proline | 41800000 | 40300000 | 52500000 | 26900000 | 40400000 | 45900000 |
| Maleic acid | 1500000 | 99800 | 433000 | 115000 | 17000 | 490000 |
| Fumarate | 24400000 | 22500000 | 28800000 | 20700000 | 27800000 | 23500000 |
| 2-Keto-isovalerate | 519000 | 187000 | 519000 | 40700 | 519000 | 468000 |
| Acetyl-glycine | 147000 | 709000 | 1990000 | 1520000 | 147000 | 1900000 |
| Indole | 416000 | 252000 | 612000 | 649000 | 799000 | 1080000 |
| Guanidoacetic acid | 656000 | 1540000 | 2160000 | 2190000 | 3770000 | 2970000 |
| Valine/5-Aminopentanoic acid | 43900 | 8140 | 125000 | 52400 | 47500 | 77100 |
| Threonine/Homoserine | 29400000 | 23800000 | 40200000 | 37800000 | 57700000 | 47600000 |
| Cysteine | 0 | 0 | 0 | 7010 | 0 | 0 |
| Taurine | 550000 | 544000 | 860000 | 323000 | 573000 | 741000 |
| Pyroglutamic acid | 259000000 | 443000000 | 482000000 | 375000000 | 369000000 | 301000000 |
| Citraconic acid | 1090000 | 1150000 | 2350000 | 1560000 | 1670000 | 3390000 |
| Ketoleucine | 1070000 | 1070000 | 178000 | 1070000 | 1070000 | 153000 |
| Hydroxyproline/Aminolevulinate | 110000 | 91700 | 249000 | 118000 | 81700 | 253000 |
| N-Acetyl-L-alanine | 790000 | 773000 | 1350000 | 1470000 | 2100000 | 1880000 |
| Oxaloacetate | 23800 | 13900 | 20700 | 14500 | 23800 | 9640 |
| Asparagine | 82400000 | 83000000 | 113000000 | 82100000 | 136000000 | 122000000 |
| Ornithine | 4180000 | 3500000 | 3580000 | 2090000 | 2770000 | 3600000 |
| Aspartate | 10100000 | 9850000 | 10800000 | 8470000 | 13600000 | 7510000 |
| Adenine | 16900000 | 17900000 | 23500000 | 25600000 | 30600000 | 32800000 |
| Methylnicotinamide | 1420000 | 1490000 | 1930000 | 2100000 | 2440000 | 2680000 |
| Anthranilate | 2510000 | 2450000 | 2940000 | 3580000 | 4280000 | 3750000 |
| p-Hydroxybenzoate | 459000 | 641000 | 543000 | 347000 | 429000 | 504000 |
| Histidinol | 170000 | 132000 | 193000 | 76600 | 38800 | 32600 |
| Alpha-ketoglutarate | 49100 | 62200 | 51500 | 44000 | 51200 | 39000 |
| Glutamine | 191000000 | 203000000 | 208000000 | 205000000 | 254000000 | 199000000 |
| Lysine | 7980000 | 7310000 | 7620000 | 10800000 | 7170000 | 7280000 |
| Glutamate | 864000000 | 991000000 | 1030000000 | 941000000 | 1170000000 | 946000000 |
| O-Acetyl-serine | 87700 | 99400 | 135000 | 90200 | 160000 | 140000 |
| 2-Oxo-4-methylthiobutanoate | 4590 | 4790 | 36600 | 6880 | 36600 | 36600 |
| Methionine | 970000 | 1870000 | 1000000 | 2480000 | 1220000 | 1280000 |
| Ribose | 2030000 | 1910000 | 1970000 | 2280000 | 1570000 | 2980000 |
| Guanine | 47800 | 65200 | 73000 | 74200 | 3280 | 33700 |
| Xanthine | 293000 | 335000 | 475000 | 345000 | 233000 | 402000 |
| Hydroxyphenylacetic acid | 49600 | 56200 | 109000 | 66700 | 44100 | 66500 |
| 2,3-Dihydroxybenzoic acid | 11100 | 12800 | 10300 | 19100 | 693 | 7350 |
| Allantoin | 38000 | 54500 | 38600 | 47100 | 74100 | 78700 |
| 2-Aminooctanoic acid | 5040000 | 2220000 | 13500 | 1400000 | 5040000 | 5040000 |
| Indole-3-carboxylic acid | 48200 | 38800 | 63800 | 19700 | 35900 | 39200 |
| Aminoadipic acid | 12200 | 14000 | 5410 | 6110 | 8500 | 24700 |
| Carnitine | 92600 | 93200 | 73900 | 59700 | 38800 | 52600 |
| Phenyllactic acid | 2750000 | 3440000 | 2260000 | 7540000 | 7010000 | 7610000 |
| Quinolinate | 21100 | 19100 | 13800 | 13700 | 11800 | 14900 |
| Phosphoenolpyruvate | 14900 | 15400 | 12700 | 12700 | 3390 | 11800 |
| 1-Methyl-histidine | 48200 | 246 | 259000 | 257000 | 246000 | 4400 |
| Dihydroxy-acetone-phosphate | 65700 | 6860 | 60600 | 70200 | 116000 | 95200 |
| sn-Glycerol-3-phosphate | 108000 | 2960 | 108000 | 108000 | 108000 | 108000 |
| Aconitate | 6360000 | 3720000 | 6360000 | 5440000 | 6360000 | 6360000 |
| N-Acetyl-L-ornithine | 944000 | 718000 | 1180000 | 1480000 | 1910000 | 1380000 |
| Acetyl-aspartate | 11700000 | 10100000 | 15300000 | 16200000 | 18400000 | 17100000 |
| Citrulline | 43900 | 44400 | 20500 | 40400 | 35100 | 22300 |
| Ascorbic acid | 8050 | 7870 | 6650 | 1460 | 18600 | 20500 |
| Allantoate | 56500 | 93800 | 45100 | 28600 | 97300 | 87300 |
| 2-Isopropylmalic acid | 1830000 | 2070000 | 1530000 | 944000 | 761000 | 831000 |
| Glucosamine | 11500 | 3510 | 4420 | 9610 | 2550 | 7050 |
| Hydroxyphenylpyruvate | 126000 | 113000 | 130000 | 387000 | 415000 | 355000 |
| 3-Phosphoglycerate | 3640 | 13500 | 2380 | 4020 | 13500 | 471 |
| N-Acetyl-glutamine | 16700000 | 28800000 | 34900000 | 51300000 | 40200000 | 33000000 |
| Acetyllysine | 76 | 76 | 76 | 76 | 76 | 76 |
| Kynurenic acid | 75600 | 38300 | 42800 | 15600 | 9100 | 13000 |
| N-Acetyl-glutamate | 1460000 | 1290000 | 1660000 | 1540000 | 1850000 | 1710000 |
| Citrate/isocitrate | 450000 | 553000 | 302000 | 173000 | 138000 | 102000 |
| 2-Keto-D-gluconate | 9840000 | 11500000 | 6540000 | 7330000 | 7160000 | 4220000 |
| Erythrose-4-phosphate | 22400 | 14200 | 10800 | 17900 | 8450 | 13000 |
| Tryptophan | 11500 | 30600 | 15200 | 1480 | 14700 | 16200 |
| Xanthurenic acid | 234000 | 226000 | 214000 | 304000 | 271000 | 338000 |
| Kynurenine | 176000 | 231000 | 153000 | 280000 | 187000 | 167000 |
| Glucarate | 2130000 | 2350000 | 1700000 | 1700000 | 1710000 | 1810000 |
| Deoxyribose-phosphate | 91000 | 91200 | 60800 | 66500 | 51800 | 71100 |
| Pantothenate | 30100000 | 34900000 | 25000000 | 40900000 | 41300000 | 34800000 |
| Cystathionine | 1070000 | 1060000 | 1710000 | 2000000 | 1790000 | 1410000 |
| Prephenate | 86400 | 70100 | 74200 | 215000 | 208000 | 185000 |
| Ribose-5-phosphate | 3810 | 9550 | 5180 | 3670 | 5180 | 5180 |
| Ribulose-5-phosphate | 60800 | 91700 | 17700 | 45200 | 43000 | 38300 |
| Pro-Asp (PD) | 341000 | 833000 | 325000 | 1460000 | 1740000 | 1010000 |
| Val-Asp (VD) | 2450000 | 2220000 | 1710000 | 2180000 | 1980000 | 1870000 |
| 5-Methoxytryptophan | 510000 | 496000 | 368000 | 562000 | 557000 | 451000 |
| Thymidine | 170000 | 134000 | 95100 | 155000 | 103000 | 98500 |
| Cytidine | 211000 | 168000 | 164000 | 133000 | 140000 | 156000 |
| Uridine | 1040000 | 1110000 | 850000 | 1250000 | 1270000 | 934000 |
| Shikimate-3-phosphate | 32100 | 31800 | 14800 | 49200 | 52300 | 17600 |
| Glucosamine-1/6-phosphate | 1850000 | 1990000 | 1450000 | 1950000 | 1440000 | 1620000 |
| Fructose-6-phosphate | 21700 | 53500 | 21200 | 21900 | 40500 | 21500 |
| Glucose-1/6-phosphate | 207000 | 191000 | 96400 | 90400 | 169000 | 159000 |
| Thiamine | 16700000 | 15200000 | 10400000 | 17900000 | 20100000 | 14100000 |
| Adenosine | 91000 | 27300 | 213000 | 56600 | 94400 | 133000 |
| 6-Phospho-D-gluconate | 44100 | 50800 | 17700 | 40900 | 35800 | 34000 |
| Xanthosine | 39000 | 60300 | 98400 | 151000 | 64400 | 68300 |
| D-Sedoheptulose-1/7-phosphate | 46700 | 37400 | 19600 | 12700 | 14400 | 2460 |
| Arginino-succinate | 63700 | 67700 | 53200 | 42100 | 54300 | 23800 |
| Glutathione | 1620000 | 1070000 | 1200000 | 1590000 | 921000 | 932000 |
| Isopentyl-pyrophosphate | 62900 | 24000 | 35900 | 16400 | 25900 | 21200 |
| UMP | 14800 | 11700 | 12500 | 73 | 11900 | 3010 |
| Cyclic-AMP | 18800 | 9850 | 11100 | 13800 | 27100 | 7920 |
| dAMP | 37400 | 14700 | 10100 | 70600 | 94900 | 27300 |
| Nicotinamide mononucleotide | 1190000 | 1160000 | 974000 | 820000 | 1080000 | 653000 |
| Trehalose/Sucrose | 12800000 | 10300000 | 16400000 | 6010000 | 11800000 | 10300000 |
| Cellobiose | 18600 | 17900 | 23700 | 12200 | 57700 | 8980 |
| Xanthosine-5-phosphate | 143000 | 104000 | 96400 | 137000 | 125000 | 138000 |
| Riboflavin | 274000 | 178000 | 219000 | 161000 | 161000 | 91300 |
| S-Adenosyl-L-homocysteine | 40000 | 36400 | 22500 | 49800 | 37100 | 26500 |
| 5-Phosphoribosyl-1-pyrophosphate | 5020 | 0 | 1780 | 1420 | 0 | 0 |
| S-Adenosyl-L-methionine | 4380 | 5240 | 14900 | 12400 | 25500 | 5530 |
| dTDP | 151000 | 110000 | 117000 | 173000 | 245000 | 152000 |
| UDP | 1920000 | 1380000 | 1850000 | 1620000 | 1140000 | 1400000 |
| Trehalose-6-Phosphate | 37300 | 23900 | 15100 | 16300 | 76600 | 34300 |
| Thiamine pyrophosphate | 30600 | 29500 | 17100 | 24700 | 22900 | 10900 |
| ADP | 0 | 4700 | 0 | 11000 | 0 | 160 |
| Adenosine 5'-phosphosulfate | 18700 | 14400 | 9440 | 13300 | 15700 | 6940 |
| Folate | 1070000 | 805000 | 626000 | 874000 | 1350000 | 801000 |
| GDP | 2170000 | 1400000 | 1930000 | 1610000 | 2570000 | 1680000 |
| CDP-ethanolamine | 29600 | 22600 | 14600 | 9870 | 40300 | 4350 |
| FMN | 203000 | 133000 | 178000 | 131000 | 260000 | 159000 |
| Cholesteryl sulfate | 6410 | 6410 | 6410 | 6410 | 6410 | 6410 |
| dUTP | 10900 | 1470 | 5180 | 0 | 1390 | 4040 |
| dTTP | 95000 | 61500 | 76700 | 79500 | 133000 | 74100 |
| CTP | 706000 | 435000 | 748000 | 374000 | 509000 | 372000 |
| UTP | 2180000 | 1510000 | 2620000 | 1390000 | 1490000 | 1140000 |
| CDP-choline | 476000 | 379000 | 406000 | 390000 | 546000 | 374000 |
| dATP | 70900 | 40500 | 57600 | 36600 | 99900 | 39000 |
| ATP | 33500000 | 20000000 | 30500000 | 17300000 | 31200000 | 17900000 |
| GTP | 1690000 | 920000 | 1650000 | 866000 | 1800000 | 955000 |
| UDP-D-glucose | 4100000 | 3130000 | 5800000 | 1940000 | 5440000 | 3330000 |
| UDP-D-glucuronate | 1010000 | 800000 | 1190000 | 777000 | 1960000 | 1050000 |
| ADP-D-glucose | 28100 | 13500 | 23800 | 14500 | 59600 | 15800 |
| Glutathione disulfide | 15200000 | 12900000 | 18300000 | 17000000 | 56900000 | 22500000 |
| NAD+ | 2140000 | 1460000 | 2410000 | 1410000 | 3650000 | 1850000 |
| FAD | 199000 | 132000 | 370000 | 162000 | 507000 | 351000 |
| Acetyl-CoA | 3070 | 0 | 23000 | 22700 | 96900 | 36000 |
| Isovaleryl/2-methylbutyryl-CoA | 40800 | 28200 | 97800 | 67000 | 281000 | 230000 |
| Hexanoyl-CoA | 10000 | 1960 | 6210 | 919 | 36700 | 12000 |
| Histidine | 17900000 | 23000000 | 16000000 | 24600000 | 22300000 | 18700000 |
