# Supplementary material for: Impact of Facultative Bacteria on the Metabolic Function of an Obligate Insect-Bacterial Symbiosis
Source: mBio. 2020 Jul 14;11(4):e00402-20. doi: 10.1128/mBio.00402-20 (PMC7360925; doi:10.1128/mBio.00402-20)
Supplement: TABLE S3 [file mBio.00402-20-st003.docx]

**TABLE S3** Differential metabolite abundance between isogenic lines bearing (SC_583) and lacking (SC_583^H-^) *Hamiltonella*.LC-MS analysis was performed on metabolite pools extracted from 7-day-old aphid larvae reared on chemically-defined diets. Benjamini-Hochberg (B-H) multiple testing corrections were performed on *p*-values obtained from *t*-tests on each metabolite.

|  |  | **Mean peak area** | |  |  |  | **95% confidence interval** | |  |
| --- | --- | --- | --- | --- | --- | --- | --- | --- | --- |
| **Metabolite** | **log2-fold enrichment in *Hamiltonella*-bearing aphids** | **SC_583** | **SC_583^H-^** | **Std. err.** | ***t*(df = 4)** | ***p*-value** | **Lower** | **Upper** | **Adjusted p-value (Benjamini-Hochberg)** |
| 5-Phosphoribosyl-1-pyrophosphate | 2.260 | 2267 | 473 | 1544 | 1.162 | 0.30997 | -2493 | 6080 | 0.72254 |
| Kynurenic acid | 2.055 | 52233 | 12567 | 11906 | 3.332 | 0.02906 | 6610 | 72723 | 0.31968 |
| D-Sedoheptulose-1-7-phosphate | 1.811 | 34567 | 9853 | 8781 | 2.814 | 0.04811 | 332 | 49095 | 0.38219 |
| Histidinol | 1.742 | 165000 | 49333 | 22481 | 5.145 | 0.00677 | 53249 | 178084 | 0.19355 |
| Maleic acid | 1.708 | 677600 | 207333 | 446221 | 1.054 | 0.35138 | -768640 | 1709173 | 0.72254 |
| dUTP | 1.692 | 5850 | 1810 | 2988 | 1.352 | 0.24771 | -4255 | 12335 | 0.65599 |
| Citrate/isocitrate | 1.660 | 435000 | 137667 | 75673 | 3.929 | 0.01711 | 87231 | 507436 | 0.27194 |
| UMP | 1.380 | 13000 | 4994 | 3675 | 2.179 | 0.09490 | -2197 | 18209 | 0.46748 |
| 2-Isopropylmalic acid | 1.098 | 1810000 | 845333 | 165052 | 5.845 | 0.00427 | 506409 | 1422924 | 0.15278 |
| Lactate | 1.046 | 30900000 | 14963333 | 8667026 | 1.839 | 0.13980 | -8126856 | 40000189 | 0.49640 |
| Isopentyl pyrophosphate | 0.951 | 40933 | 21167 | 11830 | 1.671 | 0.17007 | -13079 | 52613 | 0.50670 |
| Tryptophan | 0.823 | 19100 | 10793 | 7488 | 1.109 | 0.32951 | -12484 | 29098 | 0.72254 |
| Carnitine | 0.781 | 86567 | 50367 | 8820 | 4.104 | 0.01480 | 11712 | 60688 | 0.27194 |
| Guanine | 0.742 | 62000 | 37060 | 21850 | 1.141 | 0.31740 | -35726 | 85606 | 0.72254 |
| Riboflavin | 0.699 | 223667 | 137767 | 36239 | 2.370 | 0.07679 | -14714 | 186514 | 0.45752 |
| AICAR | 0.683 | 924000 | 575667 | 114906 | 3.031 | 0.03873 | 29302 | 667364 | 0.34611 |
| Indole-3-carboxylic acid | 0.670 | 50267 | 31600 | 9458 | 1.974 | 0.11968 | -7594 | 44927 | 0.46748 |
| UTP | 0.650 | 2103333 | 1340000 | 339084 | 2.251 | 0.08753 | -178114 | 1704781 | 0.46748 |
| Phosphoenolpyruvate | 0.625 | 14333 | 9297 | 3079 | 1.636 | 0.17717 | -3511 | 13584 | 0.50670 |
| Arginino-succinate | 0.619 | 61533 | 40067 | 9861 | 2.177 | 0.09508 | -5913 | 48847 | 0.46748 |
| CTP | 0.590 | 629667 | 418333 | 108057 | 1.956 | 0.12215 | -88680 | 511346 | 0.46748 |
| 2-Keto-D-gluconate | 0.575 | 9293333 | 6236667 | 1773130 | 1.724 | 0.15982 | -1866331 | 7979664 | 0.50670 |
| Trehalose/Sucrose | 0.491 | 13166667 | 9370000 | 2478779 | 1.532 | 0.20036 | -3085527 | 10678860 | 0.55100 |
| Ribulose-5-phosphate | 0.428 | 56733 | 42167 | 21555 | 0.676 | 0.53623 | -45279 | 74412 | 0.80418 |
| Quinolinate | 0.419 | 18000 | 13467 | 2357 | 1.923 | 0.12684 | -2012 | 11079 | 0.46748 |
| Ornithine | 0.412 | 3753333 | 2820000 | 486495 | 1.918 | 0.12749 | -417394 | 2284061 | 0.46748 |
| Ribose-5-phosphate | 0.402 | 6180 | 4677 | 1802 | 0.834 | 0.45117 | -3501 | 6508 | 0.75903 |
| Thiamine-pyrophosphate | 0.400 | 25733 | 19500 | 6123 | 1.018 | 0.36625 | -10768 | 23234 | 0.72254 |
| Nicotinamide mononucleotide | 0.381 | 1108000 | 851000 | 141415 | 1.817 | 0.14332 | -135632 | 649632 | 0.49640 |
| 2-Oxobutanoate | 0.376 | 3856000 | 2970333 | 2313971 | 0.383 | 0.72138 | -5538947 | 7310281 | 0.83867 |
| p-Hydroxybenzoate | 0.360 | 547667 | 426667 | 69435 | 1.743 | 0.15636 | -71783 | 313783 | 0.50670 |
| Deoxyribose phosphate | 0.360 | 81000 | 63133 | 11657 | 1.533 | 0.20012 | -14499 | 50232 | 0.55100 |
| Cytidine | 0.340 | 181000 | 143000 | 16513 | 2.301 | 0.08282 | -7846 | 83846 | 0.46748 |
| ATP | 0.339 | 28000000 | 22133333 | 6109919 | 0.960 | 0.39134 | -11097188 | 22830522 | 0.72677 |
| 2-3-Dihydroxybenzoic acid | 0.333 | 11400 | 9048 | 5431 | 0.433 | 0.68727 | -12727 | 17432 | 0.83867 |
| UDP | 0.308 | 1716667 | 1386667 | 219064 | 1.506 | 0.20643 | -278218 | 938218 | 0.55697 |
| CDP-ethanolamine | 0.293 | 22267 | 18173 | 11988 | 0.341 | 0.74995 | -29191 | 37378 | 0.85516 |
| Oxaloacetate | 0.285 | 19467 | 15980 | 5080 | 0.686 | 0.53019 | -10617 | 17590 | 0.80418 |
| UDP-D-glucose | 0.283 | 4343333 | 3570000 | 1282229 | 0.603 | 0.57896 | -2786705 | 4333372 | 0.80418 |
| Alpha-ketoglutarate | 0.279 | 54267 | 44733 | 5362 | 1.778 | 0.15005 | -5354 | 24421 | 0.49900 |
| Hydroxyphenylacetic acid | 0.277 | 71600 | 59100 | 20238 | 0.618 | 0.57023 | -43689 | 68689 | 0.80418 |
| Erythrose-4-phosphate | 0.269 | 15800 | 13117 | 4393 | 0.611 | 0.57432 | -9514 | 14880 | 0.80418 |
| Taurine | 0.255 | 651333 | 545667 | 160111 | 0.660 | 0.54535 | -338873 | 550206 | 0.80418 |
| 2-Keto-isovalerate | 0.253 | 408333 | 342567 | 187736 | 0.350 | 0.74377 | -455471 | 587005 | 0.85516 |
| Proline | 0.250 | 44866667 | 37733333 | 6827559 | 1.045 | 0.35510 | -11823008 | 26089675 | 0.72254 |
| Glucarate | 0.244 | 2060000 | 1740000 | 194079 | 1.649 | 0.17453 | -218850 | 858850 | 0.50670 |
| Adenosine-5-phosphosulfate | 0.243 | 14180 | 11980 | 3740 | 0.588 | 0.58799 | -8184 | 12584 | 0.80418 |
| Glucose-1,6-phosphate | 0.241 | 164800 | 139467 | 42440 | 0.597 | 0.58271 | -92500 | 143167 | 0.80418 |
| GTP | 0.234 | 1420000 | 1207000 | 388852 | 0.548 | 0.61298 | -866625 | 1292625 | 0.80418 |
| Adenosine | 0.222 | 110433 | 94667 | 58775 | 0.268 | 0.80177 | -147420 | 178953 | 0.87236 |
| 4-Aminobutyrate | 0.207 | 12966667 | 11235853 | 5949279 | 0.291 | 0.78557 | -14787033 | 18248660 | 0.87082 |
| Fructose-6-phosphate | 0.200 | 32133 | 27967 | 12387 | 0.336 | 0.75350 | -30225 | 38559 | 0.85516 |
| Pyroglutamic acid | 0.180 | 394666667 | 348333333 | 72740788 | 0.637 | 0.55878 | -155627471 | 248294138 | 0.80418 |
| Glutathione | 0.176 | 1296667 | 1147667 | 276531 | 0.539 | 0.61860 | -618772 | 916772 | 0.80418 |
| Xanthine | 0.171 | 367667 | 326667 | 74103 | 0.553 | 0.60954 | -164742 | 246742 | 0.80418 |
| Thymidine | 0.163 | 133033 | 118833 | 28221 | 0.503 | 0.64129 | -64154 | 92554 | 0.81879 |
| Citrulline | 0.154 | 36267 | 32600 | 9541 | 0.384 | 0.72031 | -22823 | 30157 | 0.83867 |
| 3-Phosphoglycerate | 0.118 | 6507 | 5997 | 5242 | 0.097 | 0.92723 | -14045 | 15065 | 0.96155 |
| Val-Asp | 0.081 | 2126667 | 2010000 | 236737 | 0.493 | 0.64797 | -540621 | 773954 | 0.82000 |
| Glucosamine-1,6-phosphate | 0.078 | 1763333 | 1670000 | 220177 | 0.424 | 0.69344 | -517975 | 704642 | 0.83867 |
| Fumarate | 0.072 | 25233333 | 24000000 | 2782884 | 0.443 | 0.68055 | -6493192 | 8959859 | 0.83867 |
| Aspartate | 0.056 | 10250000 | 9860000 | 1911683 | 0.204 | 0.84831 | -4917684 | 5697684 | 0.89197 |
| 6-Phospho-D-gluconate | 0.025 | 37533 | 36900 | 10313 | 0.061 | 0.95398 | -27999 | 29266 | 0.98143 |
| Glucosamine | 0.016 | 6477 | 6403 | 3261 | 0.022 | 0.98314 | -8981 | 9128 | 0.99932 |
| Ketoleucine | 0.016 | 772667 | 764333 | 426426 | 0.020 | 0.98534 | -1175615 | 1192282 | 0.99932 |
| Valine-5-Aminopentanoic acid | 0.000 | 59013 | 59000 | 35764 | 0.000 | 0.99972 | -99282 | 99309 | 0.99972 |
| Acetyllysine | 0.000 | 76 | 76 | 0 | NA | NA | NA | NA | NA |
| Cholesteryl sulfate | 0.000 | 6410 | 6410 | 0 | NA | NA | NA | NA | NA |
| Hydroxyproline Aminolevulinate | -0.006 | 150233 | 150900 | 71990 | -0.009 | 0.99305 | -200542 | 199209 | 0.99972 |
| Glycerate | -0.037 | 23300000 | 23900000 | 2495329 | -0.240 | 0.82180 | -7528144 | 6328144 | 0.87983 |
| dATP | -0.054 | 56333 | 58500 | 22503 | -0.096 | 0.92793 | -64645 | 60312 | 0.96155 |
| CDP-choline | -0.055 | 420333 | 436667 | 62010 | -0.263 | 0.80526 | -188500 | 155834 | 0.87236 |
| Glutamate | -0.084 | 961666667 | 1019000000 | 90629895 | -0.633 | 0.56135 | -308962260 | 194295594 | 0.80418 |
| GDP | -0.091 | 1833333 | 1953333 | 383695 | -0.313 | 0.77010 | -1185309 | 945309 | 0.86035 |
| FMN | -0.098 | 171333 | 183333 | 44207 | -0.271 | 0.79948 | -134737 | 110737 | 0.87236 |
| Allantoate | -0.126 | 65133 | 71067 | 25990 | -0.228 | 0.83061 | -78092 | 66226 | 0.87983 |
| Glutamine | -0.128 | 200666667 | 219333333 | 18135294 | -1.029 | 0.36151 | -69018315 | 31684982 | 0.72254 |
| Lysine | -0.140 | 7636667 | 8416667 | 1207707 | -0.646 | 0.55356 | -4133131 | 2573131 | 0.80418 |
| Aconitate | -0.144 | 5480000 | 6053333 | 931904 | -0.615 | 0.57168 | -3160713 | 2014046 | 0.80418 |
| Serine | -0.163 | 12460000 | 13950000 | 3557166 | -0.419 | 0.69682 | -11366277 | 8386277 | 0.83867 |
| Kynurenine | -0.179 | 186667 | 211333 | 41804 | -0.590 | 0.58687 | -140733 | 91399 | 0.80418 |
| 5-Methoxytryptophan | -0.192 | 458000 | 523333 | 57892 | -1.129 | 0.32219 | -226066 | 95400 | 0.72254 |
| S-Adenosyl-L-homocysteine | -0.197 | 32967 | 37800 | 8593 | -0.563 | 0.60380 | -28690 | 19023 | 0.80418 |
| NAD | -0.201 | 2003333 | 2303333 | 741208 | -0.405 | 0.70637 | -2357923 | 1757923 | 0.83867 |
| Uridine | -0.203 | 1000000 | 1151333 | 133698 | -1.132 | 0.32093 | -522538 | 219871 | 0.72254 |
| Histidine | -0.205 | 18966667 | 21866667 | 2704728 | -1.072 | 0.34401 | -10409530 | 4609530 | 0.72254 |
| Ribose | -0.209 | 1970000 | 2276667 | 408507 | -0.751 | 0.49458 | -1440863 | 827530 | 0.79466 |
| N-Acetylglutamate | -0.210 | 1470000 | 1700000 | 139523 | -1.648 | 0.17460 | -617378 | 157378 | 0.50670 |
| Acetoacetate | -0.212 | 1449333 | 1678667 | 992362 | -0.231 | 0.82858 | -2984572 | 2525905 | 0.87983 |
| Xanthosine-5-phosphate | -0.220 | 114467 | 133333 | 15026 | -1.256 | 0.27762 | -60587 | 22854 | 0.68447 |
| Folate | -0.274 | 833667 | 1008333 | 215085 | -0.812 | 0.46231 | -771838 | 422505 | 0.76872 |
| O-Acetyl-serine | -0.277 | 107367 | 130067 | 25159 | -0.902 | 0.41794 | -92553 | 47153 | 0.74568 |
| Asparagine | -0.289 | 92800000 | 113366667 | 19046639 | -1.080 | 0.34099 | -73448613 | 32315280 | 0.72254 |
| Cyclic AMP | -0.297 | 13250 | 16273 | 6326 | -0.478 | 0.65764 | -20587 | 14540 | 0.82494 |
| dTTP | -0.297 | 77733 | 95533 | 21146 | -0.842 | 0.44730 | -76511 | 40911 | 0.75903 |
| Thiamine | -0.301 | 14100000 | 17366667 | 2584785 | -1.264 | 0.27493 | -10443179 | 3909846 | 0.68447 |
| Aminoadipic acid | -0.315 | 10537 | 13103 | 6398 | -0.401 | 0.70881 | -20331 | 15198 | 0.83867 |
| Acetylglycine | -0.326 | 948667 | 1189000 | 762160 | -0.315 | 0.76828 | -2356430 | 1875763 | 0.86035 |
| UDP-D-glucuronate | -0.336 | 1000000 | 1262333 | 374961 | -0.700 | 0.52271 | -1303391 | 778724 | 0.80418 |
| Methionine | -0.375 | 1280000 | 1660000 | 505470 | -0.752 | 0.49400 | -1783410 | 1023410 | 0.79466 |
| Pantothenate | -0.379 | 30000000 | 39000000 | 3548709 | -2.536 | 0.06424 | -18852795 | 852795 | 0.45752 |
| Cellobiose | -0.390 | 20067 | 26293 | 15837 | -0.393 | 0.71424 | -50196 | 37743 | 0.83867 |
| Cystathionine | -0.437 | 1280000 | 1733333 | 275762 | -1.644 | 0.17554 | -1218970 | 312304 | 0.50670 |
| Xanthurenic acid | -0.438 | 224667 | 304333 | 20196 | -3.945 | 0.01689 | -135740 | -23593 | 0.27194 |
| Glycine | -0.455 | 5610000 | 7690000 | 1646410 | -1.263 | 0.27508 | -6651168 | 2491168 | 0.68447 |
| ADP-D-glucose | -0.459 | 21800 | 29967 | 15441 | -0.529 | 0.62488 | -51039 | 34706 | 0.80502 |
| Uracil | -0.470 | 355000 | 491667 | 123866 | -1.103 | 0.33179 | -480574 | 207240 | 0.72254 |
| Acetylaspartate | -0.479 | 12366667 | 17233333 | 1664999 | -2.923 | 0.04312 | -9489445 | -243888 | 0.36269 |
| Alanine/Sacrosine | -0.480 | 29833333 | 41600000 | 11661094 | -1.009 | 0.37003 | -44143054 | 20609720 | 0.72254 |
| Xanthosine | -0.521 | 65900 | 94567 | 33156 | -0.865 | 0.43603 | -120722 | 63389 | 0.75123 |
| Citraconic acid | -0.528 | 1530000 | 2206667 | 720748 | -0.939 | 0.40097 | -2677784 | 1324451 | 0.73099 |
| FAD | -0.541 | 233667 | 340000 | 122351 | -0.869 | 0.43384 | -446034 | 233368 | 0.75123 |
| Anthranilate | -0.555 | 2633333 | 3870000 | 261236 | -4.734 | 0.00908 | -1961975 | -511358 | 0.21636 |
| sn-Glycerol-3-phosphate | -0.565 | 72987 | 108000 | 35013 | -1.000 | 0.37390 | -132226 | 62199 | 0.72254 |
| Methylnicotinamide | -0.577 | 1613333 | 2406667 | 231924 | -3.421 | 0.02676 | -1437258 | -149408 | 0.31968 |
| dTDP | -0.593 | 126000 | 190000 | 30876 | -2.073 | 0.10689 | -149726 | 21726 | 0.46748 |
| Shikimate-3-phosphate | -0.598 | 26233 | 39700 | 12474 | -1.080 | 0.34107 | -48099 | 21166 | 0.72254 |
| Allantoin | -0.609 | 43700 | 66633 | 11240 | -2.040 | 0.11091 | -54141 | 8274 | 0.46748 |
| Adenine | -0.610 | 19433333 | 29666667 | 2958979 | -3.458 | 0.02586 | -18448776 | -2017891 | 0.31968 |
| Threonine/Homoserine | -0.616 | 31133333 | 47700000 | 7494517 | -2.211 | 0.09157 | -37374780 | 4241447 | 0.46748 |
| N-Acetylglutamine | -0.631 | 26800000 | 41500000 | 7545418 | -1.948 | 0.12321 | -35649439 | 6249439 | 0.46748 |
| 2-Aminooctanoic acid | -0.658 | 2424500 | 3826667 | 1894230 | -0.740 | 0.50025 | -6661393 | 3857059 | 0.79485 |
| 1-Methyl histidine | -0.723 | 102482 | 169133 | 114501 | -0.582 | 0.59173 | -384557 | 251254 | 0.80418 |
| Histamine | -0.737 | 598333 | 997000 | 166040 | -2.401 | 0.07427 | -859669 | 62336 | 0.45752 |
| Trehalose-6-Phosphate | -0.737 | 25433 | 42400 | 19002 | -0.893 | 0.42238 | -69724 | 35791 | 0.74568 |
| N-Acetyl-L-ornithine | -0.747 | 947333 | 1590000 | 210293 | -3.056 | 0.03780 | -1226533 | -58800 | 0.34611 |
| 2-Oxo-4-methylthiobutanoate | -0.800 | 15327 | 26693 | 14536 | -0.782 | 0.47792 | -51724 | 28991 | 0.78555 |
| S-Adenosyl-L-methionine | -0.825 | 8173 | 14477 | 6759 | -0.933 | 0.40383 | -25070 | 12463 | 0.73099 |
| Ascorbic acid | -0.846 | 7523 | 13520 | 6071 | -0.988 | 0.37918 | -22852 | 10859 | 0.72297 |
| N-Acetyl-L-alanine | -0.904 | 971000 | 1816667 | 264598 | -3.196 | 0.03303 | -1580309 | -111025 | 0.33733 |
| Indole | -0.982 | 426667 | 842667 | 163662 | -2.542 | 0.06386 | -870398 | 38398 | 0.45752 |
| Guanidoacetic acid | -1.036 | 1452000 | 2976667 | 631254 | -2.415 | 0.07313 | -3277309 | 227976 | 0.45752 |
| Glutathione disulfide | -1.055 | 15466667 | 32133333 | 12582351 | -1.325 | 0.25591 | -51600873 | 18267540 | 0.66537 |
| Dihydroxy acetone phosphate | -1.079 | 44387 | 93800 | 23011 | -2.147 | 0.09827 | -113303 | 14476 | 0.46748 |
| Pyruvate | -1.205 | 248333 | 572333 | 133340 | -2.430 | 0.07199 | -694211 | 46211 | 0.45752 |
| ADP | -1.248 | 1567 | 3720 | 3963 | -0.543 | 0.61575 | -13157 | 8850 | 0.80418 |
| Phenyllactic acid | -1.391 | 2816667 | 7386667 | 391181 | -11.683 | 0.00031 | -5656091 | -3483909 | 0.01463 |
| Prephenate | -1.398 | 76900 | 202667 | 10299 | -12.211 | 0.00026 | -154362 | -97171 | 0.01463 |
| Hexanoyl-CoA | -1.449 | 6057 | 16540 | 10828 | -0.968 | 0.38778 | -40545 | 19579 | 0.72677 |
| Pro/Asp | -1.490 | 499667 | 1403333 | 270204 | -3.344 | 0.02872 | -1653873 | -153460 | 0.31968 |
| dAMP | -1.632 | 20733 | 64267 | 21495 | -2.025 | 0.11282 | -103214 | 16147 | 0.46748 |
| Hydroxyphenylpyruvate | -1.649 | 123000 | 385667 | 18077 | -14.530 | 0.00013 | -312856 | -212477 | 0.01463 |
| Isovaleryl-2-methylbutyryl-CoA | -1.793 | 55600 | 192667 | 67994 | -2.016 | 0.11403 | -325849 | 51716 | 0.46748 |
| Acetyl-CoA | -2.577 | 8690 | 51867 | 23952 | -1.803 | 0.14580 | -109679 | 23326 | 0.49640 |
| Cysteine | NA | 0 | 2337 | 2337 | -1.000 | 0.37390 | -8824 | 4151 | 0.72254 |
