# Supplementary material for: Impact of Facultative Bacteria on the Metabolic Function of an Obligate Insect-Bacterial Symbiosis
Source: mBio. 2020 Jul 14;11(4):e00402-20. doi: 10.1128/mBio.00402-20 (PMC7360925; doi:10.1128/mBio.00402-20)
Supplement: TABLE S4 [file mBio.00402-20-st004.docx]

**TABLE S4** Histidine isotopes and AICAR identified in soluble metabolite and hydrolyzed protein pools by negative-mode LC-MS analysis of isogenic lines SC_583 and SC_583^H-^ reared on chemically-defined diets containing 2 mM ^13^C_6_-histidine.

| **TABLE S4A Soluble metabolite pools** | | | | | | |
| --- | --- | --- | --- | --- | --- | --- |
| **Metabolite** | **Peak area** | | | | | |
|  | SC_583 | | | SC_583^H-^ | | |
|  | Rep1 | Rep2 | Rep3 | Rep1 | Rep2 | Rep3 |
| **His M+0** | 8282485 | 10567009 | 9060396 | 10264081 | 9704522 | 8882941 |
| **His M+1** | 499983 | 674461 | 611718 | 655723 | 597578 | 569200 |
| **His M+2** | 19101 | 31099 | 28306 | 46569 | 39249 | 34277 |
| **His M+4** | 18483 | 21610 | 17754 | 30555 | 25000 | 28216 |
| **His M+5** | 573637 | 775754 | 554064 | 872385 | 876302 | 929455 |
| **His M+6** | 11754620 | 15913805 | 11454473 | 17397787 | 17511381 | 17573261 |
| **AICAR** | 1270872 | 929738 | 907813 | 596069 | 726512 | 656042 |
|  | | | | | | |
| **TABLE S4B Hydrolyzed protein pools** | | | | | | |
| **Metabolite** | **Peak area** | | | | | |
|  | SC_583 | | | SC_583^H-^ | | |
|  | Rep1 | Rep2 | Rep3 | Rep1 | Rep2 | Rep3 |
| **His M+0** | 107244397 | 152569187 | 30975836 | 90056408 | 94037833 | 107469380 |
| **His M+1** | 7089015 | 9742904 | 1872541 | 5775636 | 5416486 | 6044146 |
| **His M+2** | 140084 | 219688 | 19700 | 127156 | 159095 | 164786 |
| **His M+5** | 7207265 | 8940173 | 1985399 | 6905791 | 6870255 | 6822825 |
| **His M+6** | 133239676 | 149177289 | 38607495 | 126973241 | 131737479 | 129350583 |
