# Supplementary material for: Impact of Facultative Bacteria on the Metabolic Function of an Obligate Insect-Bacterial Symbiosis
Source: mBio. 2020 Jul 14;11(4):e00402-20. doi: 10.1128/mBio.00402-20 (PMC7360925; doi:10.1128/mBio.00402-20)
Supplement: FIG S1 [file mBio.00402-20-sf001.pdf]

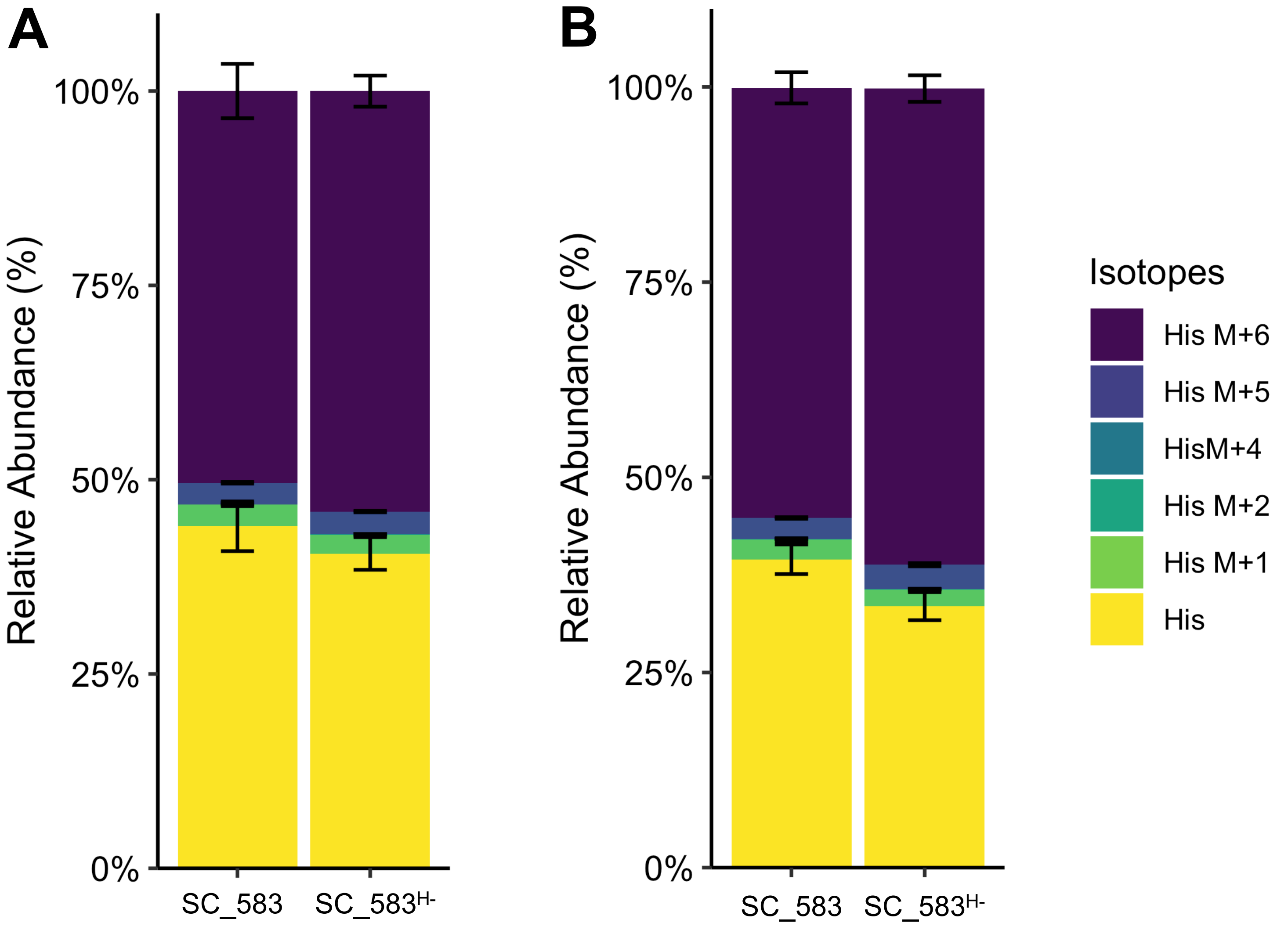

FIG S1. Relative proportions of histidine isotopes. **A** Hydrolyzed protein pools. **B** **S**oluble pools of histidine. Extracted from 7-day-old line 583 (bearing-*Hamiltonella*) and 583^{H-} (*Hamiltonella*-free) larvae reared on diets containing $^{13}C_6$-histidine from day-2 to day-7 of larval development. Error bars show standard deviation of three biological replicates per treatment and timepoint.
